# Recovery Dynamics and Prognosis After Dialysis for Acute Kidney Injury

**DOI:** 10.1001/jamanetworkopen.2024.0351

**Published:** 2024-03-08

**Authors:** Heng-Chih Pan, Hsing-Yu Chen, Nai-Chi Teng, Fang-Yu Yeh, Tao-Min Huang, Chun Yin See, Chiao-Yin Sun, Yung-Chang Chen, Likwang Chen, Vin-Cent Wu

**Affiliations:** 1 Graduate Institute of Clinical Medicine, College of Medicine, National Taiwan University, Taipei, Taiwan; 2 Chang Gung University College of Medicine, Taoyuan, Taiwan; 3 Division of Nephrology, Department of Internal Medicine, Keelung Chang Gung Memorial Hospital, Keelung, Taiwan; 4 Community Medicine Research Center, Keelung Chang Gung Memorial Hospital, Keelung, Taiwan; 5 Kidney Research Center and Department of Nephrology, Linkou Chang Gung Memorial Hospital, Taoyuan, Taiwan; 6 Division of Chinese Internal Medicine, Center for Traditional Chinese Medicine, Chang Gung Memorial Hospital, Taoyuan, Taiwan; 7 Institute of Population Health Sciences, National Health Research Institutes, Zhunan, Taiwan; 8 Division of Nephrology, Primary Aldosteronism Center of Department of Internal Medicine, National Taiwan University Hospital, Taipei, Taiwan; 9 NSARF (National Taiwan University Hospital Study Group of ARF), Taipei, Taiwan; 10 TAIPAI (Taiwan Primary Aldosteronism Investigators), Taipei, Taiwan; 11 CAKS (Taiwan Consortium for Acute Kidney Injury and Renal Diseases), Taipei, Taiwan; 12 Division of Nephrology, Department of Internal Medicine, National Cheng Kung University Hospital, College of Medicine, National Cheng Kung University, Tainan, Taiwan; 13 Division of Nephrology, Department of Internal Medicine, Linkou Chang Gung Memorial Hospital, Taoyuan, Taiwan

## Abstract

**Importance:**

The interplay among baseline kidney function, severity of acute kidney disease (AKD), and post-AKD kidney function has significant associations with patient outcomes. However, a comprehensive understanding of how these factors are collectively associated with mortality, major adverse cardiac events (MACEs), and end-stage kidney disease (ESKD) in patients with dialysis-requiring acute kidney injury (AKI-D) is yet to be fully explored.

**Objective:**

To investigate the associations of baseline kidney function, AKD severity, and post-AKD kidney function with mortality, MACEs, and ESKD in patients with AKI-D.

**Design, Setting, and Participants:**

This nationwide, population-based cohort study of patients with AKI-D was conducted between January 1, 2015, and December 31, 2018, using data from various health care settings included in the Taiwan nationwide population-based cohort database. Data analysis was conducted from April 28, 2022, to June 30, 2023.

**Exposure:**

Exposure to severe AKI and baseline and post-AKD kidney function.

**Main Outcomes and Measures:**

The primary outcomes were all-cause mortality and incident MACEs, and secondary outcomes were risks of permanent dialysis and readmission.

**Results:**

A total of 6703 of 22 232 patients (mean [SD] age, 68.0 [14.7] years; 3846 [57.4%] male) with AKI-D with post-AKD kidney function follow-up and AKD stage data were enrolled. During a mean (SD) 1.2 (0.9) years of follow-up, the all-cause mortality rate was 28.3% (n = 1899), while the incidence rates of MACEs and ESKD were 11.1% (n = 746) and 16.7% (n = 1119), respectively. After adjusting for known covariates, both post-AKD kidney function and baseline kidney function, but not AKD severity, were independently associated with all-cause mortality, MACEs, ESKD, and readmission. Moreover, worse post-AKD kidney function correlated with progressive and significant increases in the risk of adverse outcomes.

**Conclusions and Relevance:**

In this cohort study of patients with AKI-D, more than one-quarter of patients died after 1.2 years of follow-up. Baseline and post-AKD kidney functions serve as important factors associated with the long-term prognosis of patients with AKI-D. Therefore, concerted efforts to understand the transition from post-AKD to chronic kidney disease are crucial.

## Key Points

**Question:**

Are baseline kidney function, acute kidney disease (AKD) severity, and post-AKD kidney function associated with adverse outcomes in patients with acute kidney injury requiring dialysis (AKI-D)?

**Findings:**

In this cohort study with 6703 participants, baseline kidney function and post-AKD kidney function were significant independent factors associated with all-cause mortality, major adverse cardiac events, end-stage kidney disease, and readmission in patients with AKI-D. Worse post-AKD kidney function was associated with a progressive increase in the risk of adverse outcomes; however, AKD severity was not associated with adverse outcomes.

**Meaning:**

These findings suggest that evaluating baseline and post-AKD kidney function is crucial for understanding the risk of adverse outcomes in patients with AKI-D.

## Teaser

This cohort study examines the association of kidney function and acute kidney disease severity with adverse outcomes in patients with dialysis-requiring acute kidney injury.

## Introduction

Acute kidney injury (AKI) and chronic kidney disease (CKD) are conceptually interconnected syndromes.^1^ In the literature, CKD is a recognized risk factor for AKI, and the presence or absence of CKD before an episode of AKI affects the prognosis after AKI.^2^ In addition, postepisode CKD has been used as a surrogate end point for subsequent end-stage kidney disease (ESKD) and mortality in patients with AKI.^3,4^ Acute kidney disease (AKD) is an intermediate stage between AKI and CKD and is defined as acute or subacute damage and/or loss of kidney function for a duration of 7 to 90 days after an AKI episode.^5,6^ Unlike AKI and CKD, which have well-established definitions in clinical practice and public health, AKD is a relatively novel entity,^1^ and the association between the severity of AKD and outcomes is currently unclear.

Considering the increasing number of hospitalized patients who develop AKI in a wide variety of clinical settings,^7^ there is a need to monitor patients with AKI to determine when kidney function status may play a role in the assessment of adverse outcomes.^8^ We conducted this population-based cohort study to examine the associations among baseline kidney function, AKD severity, and post-AKD kidney function with adverse outcomes in patients with dialysis-requiring AKI (AKI-D).

## Methods

### Study Setting and Participants

All data for this cohort study were obtained from the National Health Insurance Research Database (NHIRD), which is a nationwide clinical database in Taiwan commonly used for various kinds of high-impact epidemiologic studies.^9,10,11^ We used NHIRD data in the Applied Health Research Data Integration Service from Taiwan’s National Health Insurance Administration (eAppendix in Supplement 1). The study flowchart is given in the Figure. The entire protocol was reviewed and approved by the institutional review board of the National Research Program for Biopharmaceuticals Institutional Review Board as well as the institutional review board of National Health Research Institutes. Because the patients’ identification numbers are encrypted and it is not possible to identify patients from the NHIRD, the need for informed consent was waived by the institutional review board. This study adheres to the Strengthening the Reporting of Observational Studies in Epidemiology (STROBE) reporting guideline (eAppendix in Supplement 1).

**Figure.**
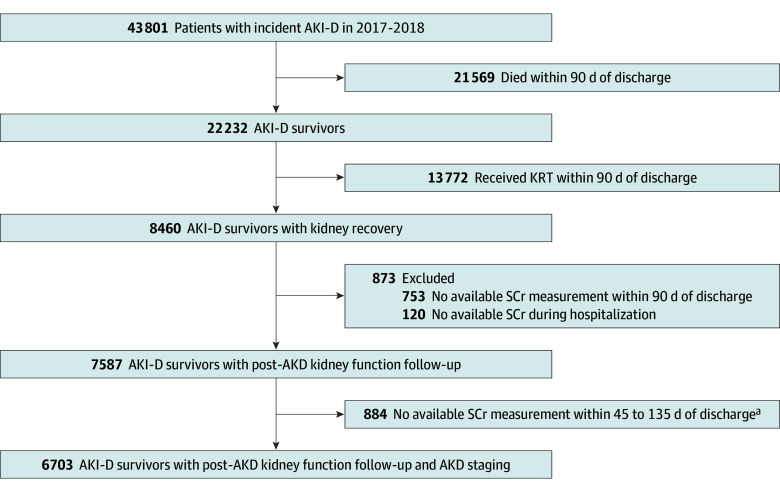
Study Flowchart AKD indicates acute kidney disease; AKI-D, dialysis-requiring acute kidney injury; KRT, kidney replacement therapy; and SCr, serum creatinine. ^a^An SCr measurement within 90 days of discharge denotes the mean value of all SCr measurements taken within 90 to 135 days after discharge.

All patients with incident AKI-D were identified between January 1, 2015, and December 31, 2018. Clinical diagnoses are recorded in the NHIRD according to *International Classification of Diseases, Ninth Revision, Clinical Modification* (*ICD-9-CM*) and *International Statistical Classification of Diseases, Tenth Revision, Clinical Modification* (*ICD-10-CM*) codes (eTable 1 in Supplement 1). Patients without a preceding diagnosis of ESKD and who received hemodialysis before the index hospitalization were defined as having AKI-D.^12^ We excluded patients younger than 18 years, patients who died or underwent additional dialysis within 90 days after discharge, and patients who did not have repeated serum creatinine (SCr) measurements during hospitalization, within 90 days after discharge, and at 90 to 180 days after discharge. In addition, patients with a history of kidney transplant were excluded.

### Measurement Definitions

Baseline SCr was defined as the lowest SCr measurement taken during outpatient visits within 180 days before the index hospitalization. If no measurement was available within this 180-day window, it was defined as the lowest SCr measurement, regardless of the clinical setting, between 180 and 360 days before the index hospitalization.^13^ If still no value was found within the 360-day timeframe, the particular sample was excluded from the study. The estimated glomerular filtration rate (eGFR) was calculated from the SCr value according to the Modification of Diet in Renal Disease equation.^14^ The baseline eGFR value was used to classify the baseline CKD stage. The eGFR value calculated as the mean value of all SCr measurements taken within 90 to 135 days after discharge was used to define the post-AKD CKD stage. The staging of AKD was determied in accordance with established guidelines.^15^ Because the SCr level may fluctuate between 7 and 90 days after AKI-D, the lowest SCr value within 90 days after discharge was used to define the presence and stage of AKD. The course of the study is shown in eFigure 1 in Supplement 1.

### Study Covariates 

*ICD-9-CM* and *ICD-10-CM* codes were used to identify comorbidities (eTable 1 in Supplement 1). Only patients with at least 2 diagnoses at outpatient department services or 1 diagnosis in a hospitalization during the study period were considered to have such comorbidities. These comorbidities were used as baseline covariates. Laboratory data were obtained within the first 7 days of hospitalization. If there were multiple test results, the first results were used. In addition, medications that were used within 180 days before the index hospitalization were defined as being coexisting medications. Data on the used medications and procedures performed during hospitalization that may have influenced kidney function are listed in eTable 2 in Supplement 1.

### Outcome Assessments

The primary outcome of this study was a composite end point that comprised all-cause mortality or incident major adverse cardiac events (MACEs). The secondary outcomes included the rates of permanent dialysis and readmission. The index date was 90 days after hospital discharge.^16^ Beginning from the index date, all eligible individuals were followed up until an event, the end of the study, or death. Coronary artery disease, heart failure, and cerebrovascular accident were classified as MACEs. We also used a subsequent selection period of 90 days to define ESKD.^9,17^

### Statistical Analysis

Data analysis was conducted from April 28, 2022, to June 30, 2023. Descriptive statistics were used to present baseline differences among the patients with different post-AKD CKD stages. Continuous data were expressed as means (SDs). Categorical data were expressed as number (percentage), and the χ^2^ or Fisher exact test was used for comparisons. All variables were tested for normal distribution using the Kolmogorov-Smirnov test. One-way analysis of variance was used to compare the means of continuous variables and normally distributed data; otherwise, the Kruskal-Wallis test was used. The variables were assessed in multivariable Cox proportional hazards regression models to estimate hazard ratios (HRs) for the possibility of all-cause mortality among 3 different scenarios: baseline CKD stages, AKD stages, and post-AKI CKD stages.^18^ Because of the high mortality rate in patients after AKD, competing risk regression analysis that took mortality into consideration was also performed using the Fine and Gray model to calculate the subdistribution HR (sHR).^19,20^ Subgroup analyses were performed to assess the differential effect of comorbidities (hypertension and diabetes), sex, and medication history (specifically, the use of angiotensin-converting enzyme inhibitors or angiotensin receptor blockers). To validate the study results, we performed a series of sensitivity analyses, including evaluating eligible cases with propensity score for multiple treatments, Cox proportional hazards regression with different covariates, and 180-day landmark analysis (excluding patients who died within 90 days and following up the remaining patients for an additional 180 days from the last day of AKD diagnosis) (eAppendix in Supplement 1). To enhance the robustness of our findings, we also adjusted the index date to the day of discharge and performed analyses that did not account for mortality as a competing risk. Specificity analyses were performed to investigate the associations between different baseline CKD stages, AKD stages, and post-AKI CKD stages and 3 independent events (deafness, appendicitis, and traffic incident), which were not attributed to subsequent exposure and observation periods, and to evaluate potentially unmeasured confounding factors. The threshold for statistical significance was set at *P* < .05, and all tests were 2-sided. All analyses were performed using R software, version 3.2.2 (R Project for Statistical Computing)^21^; SAS, version 9.2 (SAS Inc); and Stata/MP, version 16 (StataCorp LLC).

## Results

### Study Population Characteristics

Of the 22 232 patients with AKI-D, 6703 patients (mean [SD] age, 68.0 [14.7] years; 3846 [57.4%] male and 2857 [42.6%] female) with post-AKD kidney function follow-up and AKD stage information were enrolled. Compared with the excluded patients, the enrolled patients were older, had a lower prevalence of hypertension and diabetes, and exhibited better kidney function (eTable 3 in Supplement 1). During a mean (SD) follow-up period of 1.2 (0.9) years, the overall mortality rate was 28.3% (n = 1899 of 6703), 746 patients (11.1%) developed MACEs, and 1119 patients (16.7%) developed ESKD (Table 1). The mean (SD) times to adverse outcomes were as follows: all-cause mortality, 429.5 (321.6) days; MACE development, 393.6 (315.5) days; ESKD development, 367.7 (304.7) days; and readmission, 203.4 (243.8) days. The mean (SD) eGFR was 52.9 (43.8) mL/min/1.73 m^2^. Acute kidney injury was associated with sepsis in 2803 patients (41.8%). Overall, 4527 patients (67.5%) had hypertension and 2750 (41.0%) had diabetes. The patients with post-AKD CKD stage 5 were older and had a higher Charlson Comorbidity Index score and lower baseline eGFR than those with post-AKD CKD stages 1 to 4. The prevalence of comorbidities was significantly different among the patients with different post-AKD kidney function. Specifically, the patients with post-AKD CKD stage 5 were associated with higher prevalence rates of hypertension, diabetes, myocardial infarction, congestive heart failure, hyperlipidemia, hyperuricemia, and cerebrovascular disease but lower prevalence rates of male sex and malignant tumors than the patients with post-AKD CKD stages 1 to 4 (Table 1).

**Table 1.** Baseline Characteristics of Enrollees According to Post-AKD CKD Stages^a^

**Characteristic**	Total patients (N = 6703)	Stages 0-2 (n = 1572)	Stage 3 (n = 1818)	Stage 4 (n = 1483)	Stage 5 (n = 1830)	*P* value^b^
Demographic factors						
Age, mean (SD), y	68.0 (14.7)	60.3 (16.5)	69.2 (13.9)	72.4 (12.4)	69.7 (13.1)	<.001
Sex						
Male	3846 (57.4)	1046 (66.5)	1088 (59.8)	813 (54.8)	899 (49.1)	<.001
Female	2857 (42.6)	526 (33.5)	730 (40.2)	670 (45.2)	931 (50.9)	
CCI score, mean (SD)	4.2 (2.5)	3.0 (2.6)	4.0 (2.5)	4.9 (2.4)	4.9 (2.1)	<.001
Hypertension	4527 (67.5)	737 (46.9)	1200 (66.0)	1155 (77.6)	1440 (78.7)	<.001
Diabetes	2750 (41.0)	448 (28.5)	749 (41.2)	718 (48.4)	835 (45.6)	<.001
Myocardial infarction	684 (10.2)	92 (5.9)	191 (10.5)	189 (12.7)	212 (11.6)	<.001
Congestive heart failure	2042 (30.5)	266 (16.9)	547 (30.1)	593 (40.0)	636 (34.8)	<.001
Hyperlipidemia	1945 (29.0)	359 (22.8)	545 (30.0)	464 (31.3)	577 (31.5)	<.001
Hyperuricemia	828 (12.4)	132 (8.4)	197 (10.8)	222 (15.0)	277 (15.1)	<.001
Cerebrovascular disease	1125 (16.8)	250 (15.9)	291 (16.0)	310 (20.9)	274 (15.0)	<.001
Malignant tumors	1190 (17.8)	321 (20.4)	360 (19.8)	261 (17.6)	248 (13.6)	<.001
COPD	961 (14.3)	206 (13.1)	286 (15.7)	242 (16.3)	227 (12.4)	.002
Baseline eGFR, mean (SD), mL/min/1.73 m^2^	52.9 ( 43.8)	95.3 (50.7)	61.4 (33.1)	37.5 (25.4)	20.7 (19.2)	<.001
Baseline kidney function						
CKD stages 0-2	2305 (34.4)	1266 (80.5)	784 (43.1)	175 (11.8)	80 (4.4)	<.001
CKD stage 3	1856 (27.7)	230 (14.6)	844 (46.4)	603 (40.7)	179 (9.8)	
CKD stage 4	1512 (22.6)	42 (2.7)	147 (8.1)	628 (42.4)	695 (38.0)	
CKD stage 5	1030 (15.4)	34 (2.2)	43 (2.4)	77 (5.2)	876 (47.9)	
AKD severity						
AKD stage 0	5543 (82.7)	1477 (94.0)	1521 (83.7)	1177 (79.4)	1368 (74.8)	<.001
AKD stage 1	763 (11.4)	75 (4.8)	211 (11.6)	184 (12.4)	293 (16.0)	
AKD stage 2	288 (4.3)	15 (1.0)	75 (4.1)	83 (5.6)	115 (6.3)	
AKD stage 3	109 (0.5)	NA	11 (0.6)	39 (2.6)	54 (3.0)	
Intervention during index hospitalization						
Hospitalization, mean (SD), d	28.4 (27.7)	31.1 (20.9)	29.7 (27.0)	29.5 (28.4)	24.1 (32.0)	<.001
ICU admission	4875 (72.7)	1363 (86.7)	1421 (78.2)	1045 (70.5)	1046 (57.2)	<.001
Oxygen therapy	6098 (91.0)	1490 (94.8)	1687 (92.8)	1352 (91.2)	1569 (85.7)	<.001
MV	2929 (43.7)	1034 (65.8)	902 (49.6)	553 (37.3)	440 (24.0)	<.001
Prolonged MV >24 h	2749 (41.0)	1001 (63.7)	847 (46.6)	515 (34.7)	386 (21.1)	<.001
ARDS	328 (4.9)	138 (8.8)	102 (5.6)	43 (2.9)	45 (2.5)	<.001
CABG	135 (2.0)	24 (1.5)	42 (2.3)	35 (2.4)	34 (1.9)	.28
PTCA	432 (6.4)	42 (2.7)	85 (4.7)	120 (8.1)	185 (10.1)	<.001
IABP	187 (2.8)	55 (3.5)	63 (3.5)	40 (2.7)	29 (1.6)	.001
ECMO	158 (2.4)	78 (5.0)	58 (3.2)	15 (1.0)	7.0 (0.4)	<.001
Major operation						
Cardiac surgery	476 (7.1)	158 (10.1)	171 (9.4)	85 (5.7)	62 (3.4)	<.001
Thoracic surgery	266 (4.0)	90 (5.7)	86 (4.7)	59 (4.0)	31 (1.7)	<.001
Aorta surgery	93 (1.4)	35 (2.2)	35 (1.9)	16 (1.1)	7 (0.4)	<.001
Esophagus surgery	52 (0.8)	26 (1.7)	13 (0.7)	8 (0.6)	N/A	<.001
Gastric surgery	64 (1.0)	27 (1.7)	15 (0.8)	13 (0.9)	9 (0.5)	.003
Intestine surgery	122 (1.8)	46 (2.9)	38 (2.1)	23 (1.6)	15 (0.8)	<.001
Rectum surgery	30 (0.5)	10 (0.6)	10 (0.6)	NA	7 (0.4)	.11
Liver surgery	111 (1.7)	51 (3.2)	29 (1.6)	19 (1.3)	12 (0.7)	<.001
Biliary surgery	52 (0.8)	22 (1.4)	9 (0.5)	11 (0.7)	10 (0.6)	.01
Pancreas surgery	17 (0.3)	12 (0.8)	NA	0 (0)	0 (0)	<.001
AKI contributor						
Sepsis dominant	2803 (41.8)	879 (55.9)	853 (46.9)	594 (40.1)	477 (26.1)	<.001
Hypovolemic shock dominant	165 (2.5)	63 (4.0)	47 (2.6)	37 (2.5)	18 (1.0)	<.001
Contrast dominant	1663 (24.8)	510 (32.4)	447 (24.6)	357 (24.1)	349 (19.1)	<.001
Other or mixed causes^c^	4114 (61.4)	392 (24.5)	1018 (56.0)	1088 (73.4)	1622 (88.7)	<.001
Medication before index hospitalization						
Antiplatelet	836 (12.5)	157 (10.0)	266 (14.6)	206 (13.9)	207 (11.3)	<.001
Statin	2448 (36.5)	386 (24.6)	695 (38.2)	597 (40.3)	770 (42.1)	<.001
Urate-lowering drug	2237 (33.4)	258 (16.4)	530 (29.2)	621 (41.9)	828 (45.3)	<.001
α-Blocker	107 (1.6)	8 (0.5)	26 (1.4)	36 (2.4)	37 (2.0)	<.001
β-Blocker	718 (10.7)	96 (6.1)	161 (8.9)	169 (11.4)	292 (16.0)	<.001
ACEI or ARB	449 (6.7)	74 (4.7)	116 (6.4)	111 (7.5)	148 (8.1)	<.001
MRA	248 (3.7)	49 (3.1)	74 (4.1)	70 (4.7)	55 (3.0)	.03
CCB	928 (13.8)	132 (8.4)	235 (12.9)	220 (14.8)	341 (18.6)	<.001
Other antihypertensives	136 (2.0)	9 (0.6)	33 (1.8)	32 (2.2)	62 (3.4)	<.001
Discharge BUN, mean (SD), mg/dL	37.0 (25.2)	16.5 (9.1)	25.8 (13.4)	39.9 (18.5)	60.4 (25.2)	<.001
Discharge eGFR, mean (SD), mL/min/1.73 m^2^	55.5 (61.4)	123.4 (81.5)	57.4 (38.1)	30.2 ( 15.0)	15.9 (20.9)	<.001
AKD ratio, mean (SD)	1.16 (0.61)	0.88 (0.41)	1.12 (0.51)	1.25 (0.67)	1.37 (0.69)	<.001
Outcome						<.001
All-cause mortality	1899 (28.3)	328 (20.9)	523 (28.8)	534 (36.0)	514 (28.1)	<.001
MACE	746 (11.1)	82 (5.2)	182 (10.0)	185 (12.5)	297 (16.2)	<.001
ESKD	1119 (16.7)	NA	32 (1.8)	186 (12.5)	900 (49.2)	<.001
Readmission	4333 (64.6)	869 (55.3)	1093 (60.1)	993 (67.0)	1378 (75.3)	<.001

Abbreviations: ACEI, angiotensin-converting enzyme inhibitor; AKD, acute kidney disease; AKI, acute kidney injury; ARB, angiotensin receptor blocker; ARDS, acute respiratory distress syndrome; BUN, blood urea nitrogen; CABG, coronary artery bypass graft; CCB, calcium channel blocker; CCI, Charlson Comorbidity Index; CKD, chronic kidney disease; COPD, chronic obstructive pulmonary disease; ECMO, extracorporeal membrane oxygenation; eGFR, estimated glomerular filtration rate; ESKD, end-stage kidney disease; IABP, intra-aortic balloon pump; ICU, intensive care unit; MACE, major adverse cardiac event; MRA, mineralocorticoid receptor antagonist; MV, mechanical ventilation; NA, not applicable; and PTCA, percutaneous transluminal coronary angioplasty.

SI conversion factor: To convert BUN to millimoles per liter, multiply by 0.357.

^a^ Data are presented as number (percentage) of patients unless otherwise indicated.

^b^ Analysis of variance.

^c^ Other or mixed causes include nephrotoxic agent, cardiogenic shock, cardiorenal syndrome, obstructive uropathy, hypertension crisis, and postpartum hemorrhage.

Before the index hospitalization, the patients with post-AKD CKD stage 5 were more likely to use statins, urate-lowering agents, α-blockers, β-blockers, and calcium channel blockers than the patients with post-AKD CKD stages 1 to 4. During the index hospitalization, the patients with post-AKD CKD stage 5 were more likely to have a shorter hospital stay and less likely to be admitted to an intensive care unit than the patients with post-AKD CKD stages 1 to 4.

### Post-AKD Kidney Function and Adverse Outcomes in Patients With AKI-D 

In multivariable analysis, baseline kidney function and post-AKD kidney function, but not AKD severity, were associated with all-cause mortality, MACEs, ESKD, and readmission (eTable 4 in Supplement 1 and Table 2). Worse post-AKD kidney function was associated with a progressive and significant increase in the risk of adverse outcomes. The HRs for all-cause mortality calculated as per the stratification data of post-AKD kidney function were 1.19 (95% CI, 1.02-1.38) for post-AKD CKD stage 3, 1.58 (95% CI, 1.32-1.89) for stage 4, and 1.56 (95% CI, 1.26-1.93) for stage 5. Taking mortality as a competing risk, the sHRs for incident MACEs, ESKD, and readmission increased with the severity of post-AKD CKD stage. Worse baseline kidney function was also associated with an increased sHR for ESKD. However, this trend was not observed in the associations between the other outcomes of interest and baseline kidney function.

**Table 2.** Cox Proportional Hazards Regression Models Depicting the Possibility of All-Cause Mortality, MACE, ESKD, and Readmission

Factor	All-cause mortality		MACE^a^		ESKD^a^		Readmission^a^	
	HR (95% CI)	*P* value	sHR (95% CI)	*P* value	sHR (95% CI)	*P* value	sHR (95% CI)	*P* value
Baseline kidney function^b^								
CKD stage 3	0.84 (0.73-0.97)	.02	1.36 (1.06-1.74)	.02	2.65 (1.76-3.97)	<.001	0.93 (0.84-1.02)	.12
CKD stage 4	0.72 (0.60-0.86)	<.001	1.59 (1.19-2.11)	.001	4.90 (3.30-7.28)	<.001	0.90 (0.80-1.02)	.09
CKD stage 5	0.57 (0.45-0.71)	<.001	1.38 (0.99-1.92)	.055	8.44 (5.62-12.69)	<.001	0.86 (0.74-0.99)	.04
AKD severity^c^								
AKD stage 1	0.92 (0.80-1.07)	.30	0.96 (0.76-1.21)	.73	1.75 (1.47-2.08)	<.001	1.00 (0.91-1.10)	.97
AKD stage 2	1.01 (0.81-1.27)	.93	1.01 (0.70-1.47)	.94	1.97 (1.49-2.59)	<.001	1.01 (0.87-1.17)	.93
AKD stage 3	0.73 (0.49-1.09)	.12	0.57 (0.25-1.34)	.20	1.96 (1.17-3.27)	.01	1.04 (0.83-1.29)	.76
Post-AKD kidney function^b^								
CKD stage 3	1.19 (1.02-1.38)	.03	1.49 (1.11-2.01)	.008	18.22 (2.49-133.41)	.004	1.06 (0.95-1.17)	.28
CKD stage 4	1.58 (1.32-1.89)	<.001	1.53 (1.09-2.13)	.01	79.09 (11.13-562.04)	<.001	1.23 (1.09-1.40)	.001
CKD stage 5	1.56 (1.26-1.93)	<.001	1.99 (1.39-2.83)	<.001	253.42 (35.83-1729.38)	<.001	1.65 (1.43-1.90)	<.001

Abbreviations: AKD, acute kidney disease; CKD, chronic kidney disease; ESKD, end-stage kidney disease; HR, hazard ratio; MACE, major adverse cardiac event; sHR, subdistribution hazard ratio.

^a^ Taking mortality as a competing risk.

^b^ Compared with the risk of an estimated glomerular filtration rate greater than 60 mL/min/1.73 m^2^.

^c^ Compared with the risk of non-AKD.

eFigure 2 in Supplement 1 depicts the HRs and sHRs for adverse outcomes according to the stage of AKD, baseline CKD, and post-AKD CKD. The HRs and SHRs for adverse outcomes in most stages of AKD and baseline CKD were approximately 1.0 (reference: AKD stage 0, baseline CKD stages 0-2, and post-AKD CKD stages 0-2) except for the SHRs for MACEs and ESKD in the stages of baseline CKD. In contrast, the HRs and SHRs for adverse outcomes in the post-AKD CKD stages were all greater than 1.0, depicting an increase with severity.

### Association Between Changes in Kidney Function and Long-Term Outcomes

We observed that the correlation between baseline kidney function and post-AKD kidney function was high and significant; however, the correlation of AKD severity with the other 2 exposures of interest was weak (eTable 5 in Supplement 1). eTable 6 in Supplement 1 gives the population distribution of patients with different baseline CKD stages who had different post-AKD CKD stages. A total of 3614 patients (53.9%) with AKI-D in this study returned to their initial CKD stage after AKD. However, there was a trend toward no kidney recovery in the patients with advanced baseline CKD. The prevalence of post-AKD CKD stage 5 increased with increasing baseline CKD stage (3.5%, 9.6%, and 46.0% for patients with baseline CKD stages 0-2, 3, and 4, respectively).

Table 3 details the interaction of baseline kidney function and post-AKD kidney function with the risk of the composite of mortality or MACEs. The risk of mortality or MACEs increased with increasing post-AKD CKD stage in the patients with the same baseline CKD stage. We also examined the interaction of baseline kidney function and post-AKD kidney function on the risk of ESKD. Compared with the patients with baseline CKD stages 0 to 2 who had complete kidney recovery after AKD, the risk of ESKD increased with increasing baseline CKD stage in those with post-AKD CKD stage 5 after adjusting for all covariates (Table 4).

**Table 3.** Risk of Mortality or Major Adverse Cardiac Event According to the Combination of Baseline Kidney Function and Post-AKD Kidney Function

Post-AKD kidney function by baseline kidney function	No (%) of patients	HR (95% CI)^a^	*P* value
**Baseline CKD stages 0-2 (n = 659)**			
CKD stages 0-2	301 (45.7)	1 [Reference]	NA
CKD stage 3	260 (39.5)	1.29 (1.10-1.51)	.002
CKD stage 4	69 (10.5)	1.71 (1.30-2.25)	<.001
CKD stage 5	29 (4.4)	1.70 (1.15-2.52)	.008
Total	659 (100)	NA	NA
**Baseline CKD stage 3 (n = 707)**			
CKD stage 3	302 (42.7)	1.15 (0.98-1.34)	.08
CKD stage 4	257 (36.4)	1.39 (1.18-1.64)	<.001
CKD stage 5	83 (11.7)	1.79 (1.39-2.31)	<.001
Total	642 (90.8)	NA	NA
**Baseline CKD stage 4 (n = 633)**			
CKD stage 4	274 (43.3)	1.35 (1.15-1.59)	<.001
CKD stage 5	297 (46.9)	1.46 (1.24-1.72)	<.001
Total	571 (90.2)	NA	NA
**Baseline CKD stage 5 (n = 339)**			
CKD stage 5	302 (89.1)	1.21 (1.02-1.42)	.03
**Restore to milder CKD stages (n = 164)**			
NA	NA	1.24 (0.83-1.86)	.29

Abbreviations: AKD, acute kidney disease; CKD chronic kidney disease; HR, hazard ratio; NA, not applicable.

^a^ The HRs were adjusted for age, sex, length of stays, Charlson Comorbidity Index scores, AKD stage, comorbidity, drug use, and medical procedures.

**Table 4.** Risk of End-Stage Kidney Disease According to the Combination of Baseline Kidney Function and Post-AKD Kidney Function

Post-AKD kidney function by baseline kidney function	No (%) of patients	HR (95% CI)^a^	*P* value
**Baseline CKD stages 0-2 (n = 31)**			
CKD stages 0-2	NR^b^	1 [Reference]	NA
CKD stage 3	13 (41.9)	11.98 (2.69-53.39)	.001
CKD stage 4	10 (32.3)	31.98 (6.91-148.00)	<.001
CKD stage 5	7 (22.6)	45.89 (9.55-220.60)	<.001
Total	31 (100)	NA	NA
**Baseline CKD stage 3 (n = 136)**			
CKD stage 3	18 (13.2)	19.14 (4.43-82.76)	<.001
CKD stage 4	58 (42.6)	73.60 (17.86-303.20)	<.001
CKD stage 5	60 (44.1)	269.22 (65.12-1113.03)	<.001
Total	136 (100)	NA	NA
**Baseline CKD stage 4 (n = 432)**			
CKD stage 4	110 (25.5)	167.62 (41.12-683.22)	<.001
CKD stage 5	321 (74.3)	459.75 (113.53-1861.82)	<.001
Total	431 (99.8)	NA	NA
**Baseline CKD stage 5 (n = 520)**			
CKD stage 5	512 (98.5)	837.35 (207.52-3378.79)	<.001
**Restore to milder CKD stages (n = 9)**			
NA	NA	117.25 (24.78-554.84)	<.001

Abbreviations: AKD, acute kidney disease; CKD chronic kidney disease; HR, hazard ratio; NA, not applicable; NR, not reported.

^a^ The HRs were adjusted for age, sex, length of stays, Charlson Comorbidity Index scores, AKD stage, comorbidity, drug use, and medical procedures.

^b^ According to the requirements of Taiwan’s National Health Insurance Administration, to prevent the leakage of personal information, data regarding the number of patients cannot be reported when using the National Health Insurance database if the count is less than 5.

eFigure 3 in Supplement 1 illustrates the natural course of kidney function in the patients who had an AKI episode. Sankey diagrams show the flow of changes in kidney function and outcomes with proportional arrow magnitudes and demonstrate that the severity of AKD was not associated with the distribution of post-AKD kidney function or further long-term outcomes as in our Cox proportional hazards regression model. These results implied that the patients who did not progress to AKD remained at risk of adverse outcomes. Compared with the progression of baseline CKD to AKD to adverse outcomes, the progression of baseline CKD to post-AKD CKD to adverse outcomes appears to be a more coherent and natural dynamic process.

### Subgroup and Sensitivity Analyses

In subgroups analyses, the association between the patients with poor post-AKD kidney function (post-AKD CKD stages 3-5) and a higher risk of death than those without CKD after AKD (post-AKD CKD stages 0-2) remained consistent across age, sex, diabetes, hypertension, congestive heart failure, cerebrovascular disease, and use of renin-angiotensin-aldosterone system inhibitors. There were no significant interactions between post-AKD kidney function and stratified covariates except for diabetes (eFigure 4 in Supplement 1). In other words, patients with diabetes and advanced post-AKD CKD had a higher risk of mortality than patients without diabetes. A series of sensitivity analyses were also conducted. Different models were used to evaluate the results, including eligible cases with propensity scores for multiple treatments, Cox proportional hazards regression analysis with different covariates, and overlap weighting with different sampling populations. eFigure 5 in Supplement 1 illustrates the influence of overlap weighting on covariate balance and treatment effect estimation. In all models, there was a similar trend toward an increased risk of adverse events with worse post-AKD kidney function (eTables 7-8 in Supplement 1). We also performed additional analyses by setting the index date as the day of discharge and not taking mortality as a competing risk (eTables 9-11 in Supplement 1). The outcomes of these sensitivity analyses corroborated our primary analysis, reinforcing the robustness of the association between post-AKD kidney function and mortality risk. Furthermore, our specificity analysis indicated that the prevalence rates of deafness, appendicitis, and traffic incidences did not significantly differ across the different baseline CKD stages, AKD stages, or post-AKD CKD stages (eTables 12-14 in Supplement 1).

## Discussion

In this study, of the patients with AKI-D who experienced kidney function recovery and were subsequently weaned off kidney replacement therapy, more than one-quarter died, 16.7% developed ESKD, and 11.1% developed incident MACEs after a mean of 1.2 years of follow-up. Nearly half of the patients with AKI-D in this study returned to their initial CKD stage after AKD. A significant observation from our data is that almost 50% of patients with AKI-D died within 90 days after discharge (Figure). Among these patients, approximately 37% died during their hospitalization, while the remaining 12% died within 90 days after discharge. This distinction underscores the critical impact of AKI-D on patient outcomes, both during and after their hospital stay. Furthermore, the risk of needing additional dialysis within this 90-day window was alarmingly high, affecting more than half of the patients with AKI-D. Our results revealed that baseline kidney function alongside post-AKD kidney function, but not AKD severity, were significantly associated with all-cause mortality, incident MACEs, ESKD, and readmission. Notably, the severity of post-AKD kidney function was associated with progressive and significant increases in adverse outcomes (eFigure 6 in Supplement 1).

Sawhney et al^22^ reported that the outcomes of patients with AKI with good postepisode kidney function were significantly better than in those with poor postepisode kidney function, which is consistent with our findings. In our study, the patients who did not return to their initial CKD stage after AKD had a much higher incidence of mortality or MACEs than the patients without CKD after AKD, supporting the theory that kidney recovery is an independent protective factor for mortality in hospitalized patients with AKI.^23,24^ Few previous studies have examined in detail how post-AKD kidney function affects the long-term prognosis of patients with AKI. To the best of our knowledge, this is the first study to demonstrate that worse post-AKD kidney function was associated with progressive increases in the risk of mortality, incident MACEs, ESKD, and readmission.

Compared with the severity of AKD, post-AKD kidney impairment may signify longer persistent inflammation, maladaptive kidney repair, greater kidney damage, or repeated kidney tissue injury. All of these factors may, at least partially, explain why post-AKD kidney function was associated with adverse outcomes in patients with AKI-D.^25^ Kidney dysfunction impacts innate and adaptive immunity, autoregulation, and vasodilation response and reduces tolerance to adverse effects of drugs.^3,26^ Patients with kidney impairment are susceptible to infections, which can contribute to septic shock, metabolic acidosis, and AKI.^3,27^ A previous study proposed that pre-AKI kidney function should be considered as an additional factor that modifies the subsequent risk of mortality and CKD after AKI.^2^ Our study focused on patients who survived AKI-D, the most severe form of AKI. A total of 4398 of 6703 patients (65.6%) had significant baseline kidney impairment (CKD stages 3-5), and sepsis was the leading dominant cause of AKI (41.8%). Poorer baseline kidney function was associated with an increased risk of MACEs and ESKD after follow-up, which is in line with previous studies.^2^ In contrast, the association between baseline kidney function and readmission was not significant (baseline CKD stages 3-5) in our study. Furthermore, our model also suggested an association between higher baseline eGFR values and lower risk of mortality, which is consistent with a recent study.^25^ A possible explanation is that patients with lower baseline eGFR values would have greater fluctuations in SCr and be more susceptible to fluid overload, uremia, and acid-base or electrolyte disturbances and may be eligible for dialysis despite less damage.^3,28^ Moreover, patients with AKI-D have a poor prognosis,^29^ which may have reduced the impact of poor baseline kidney function contributing to the adverse outcomes.

Several previous studies^30,31^ have reported that the severity of AKD was correlated with higher risks of long-term adverse kidney events and mortality. However, conflicting results were also reported in some cohorts of patients with AKD.^30,32^ Furthermore, some of the studies were conducted at a single center, so accurate information about possible adverse outcomes in other health care systems is not available.^30,31^ Most of our patients with AKI had temporary complete kidney function recovery (SCr returned to lower than 1.5 times the baseline level) within 7 to 90 days after the episode. Nevertheless, 1899 of 6703 patients (28.3%) died, and 1119 patients (16.7%) developed ESKD in long-term follow-up.

The discrepancy in AKD stages not aligning with long-term outcomes can be attributed to the limitations of SCr as an acute illness kidney function marker. Haines et al^33^ and Lumlertgul et al^34^ have indicated that SCr values in the initial 3 to 6 months after discharge may not be as trustworthy. This unreliability is possibly due to the impact of acute skeletal muscle wasting on SCr, which may lead to kidney function overestimation. Furthermore, post-AKI maladaptive repair processes, such as tubulointerstitial fibrosis, can mask genuine recovery but eventually result in a continuous decline in kidney function.^35^ These findings suggest that AKD status cannot signal progression after AKI-D or provide adequate information for risk stratification of patients with AKI-D.

Of note, the association between poor post-AKD kidney function and higher risk of mortality was more significant in patients who had diabetes. Considering that diabetes is a major cause of CKD,^36^ our results have an important clinical implication and may provide valuable information to physicians when making a risk assessment. Further management to reduce the severity of post-AKD CKD is important. In addition, our study underscores the importance of paying attention to individuals with initially healthy kidney function who undergo kidney function recovery and are subsequently weaned off dialysis. This nuanced perspective should be thoughtfully incorporated into decision-making processes. Building on the recommendations of the Acute Disease Quality Initiative 22 Working Group, which emphasized the importance of kidney health assessments in patients with AKD,^37^ we suggest that future strategies should incorporate baseline kidney function and post-AKD kidney function as stratifying risk factors for adverse outcomes.

### Limitations

This study has several potential limitations that should be recognized. First, because this study is retrospective, the kidney parameters were not regularly measured, which could have led to healthy survivor bias. However, we used routinely collected data from a nationwide, population-based database with long follow-up, which maximizes the clinical implication of our findings. Second, we applied overlap weighting, inspired by the principles of a randomized clinical trial, to minimize the effect of extreme treatment propensities.^38^ This method has been recognized for its superiority over inverse probability weighting in bias, variance, and coverage for time-to-event outcomes, particularly when there is reduced covariate overlap between treatment groups.^39^ However, we acknowledge its potential incomparability with our main analysis. Third, patients who died during hospitalization or within 90 days after discharge were excluded from our cohort. As a result, we lack data on the post-AKD CKD stages for these individuals, making it impossible to determine the intensive care unit admission ratios based on post-AKD CKD stages for this group. Additionally, our conclusions and findings are specific to the selected cohort and may not accurately reflect the likelihood of intensive care unit admissions, the overall disease prevalence, and the incidence of major surgery among all patients with AKD, especially those who experienced mortality during or shortly after their hospital stay. Nevertheless, we conducted sensitivity analyses that included all discharged patients, and the results were consistent with our primary analysis. Fourth, our study design inherently focuses on 3 exposures: baseline CKD, AKD, and post-AKD CKD within a single cohort. This focus means that although we are examining the associations among these 3 exposures, we cannot completely isolate and evaluate each exposure independently. Only patients with data for all 3 exposures were included in our analysis, which may introduce a potential bias. Furthermore, we estimated the association between the post-AKD kidney function and long adverse outcomes, which may lead to an immortal time bias.^40^ However, we could focus on the prognosis of the patients with AKI-D after discharge, allowing for a more accurate analysis of adverse outcomes in patients with severe AKI and limited immortal time bias.

## Conclusions

This study provides clinical evidence demonstrating that post-AKD kidney function could be considered an independent factor associated with the long-term prognosis of patients with AKI-D. On the basis of our results, we suggest that stratifying patients with AKI-D by post-AKD and baseline kidney function can improve the prognostic prediction along with objective information in clinical practice. Further coordination of care to reduce the post-AKD CKD transition is important.

## Notes

Supplement 1. **eAppendix.** Supplementary Material and Methods **eFigure 1.** Time Course of the Study **eFigure 2.** The Risk for the Adverse Outcomes in the Stages of AKD, Baseline CKD, and Post-AKD CKD **eFigure 3.** Sankey Diagrams Showing the Natural Course of Kidney Function in the Patients Who Had an AKI Episode **eFigure 4.** Forest Plots of Subgroup Analyses According to Baseline Characteristics for Stage 3-5 vs. Stage 0-2 After AKD **eFigure 5.** The Influence of Overlap Weighting on Covariate Balance and Treatment Effect Estimation **eFigure 6.** Graphic Abstract **eTable 1.** Diagnosis Codes Used in the Study **eTable 2.** Medication Codes Used in This Study **eTable 3.** Baseline Characteristics of Enrolled Patients and Excluded Patients **eTable 4.** Cox Proportional Hazards Models for Adverse Outcomes, Fitting Models With Respect to Baseline Kidney Function, AKD Severity, and Post-AKD Kidney Function (Separate Models for Each Exposure) **eTable 5.** Correlation Between Baseline Kidney Function, AKD Severity, and Post-AKD Kidney Function **eTable 6.** Patient Distribution According to the Combination of Baseline Kidney Function and Post-AKD Kidney Function **eTable 7.** Sensitivity Analysis for Risk of Mortality Among Different Post-AKI-CKD Stages **eTable 8.** Sensitivity Analysis for Risk of MACE or Mortality Among Different Post-AKI-CKD Stages **eTable 9.** Sensitivity Analysis for Risk of All-Cause Mortality, MACE, ESKD, and Readmission Across Different Post-AKI-CKD Stages With Discharge Date as the Time Zero Point **eTable 10.** Cause-Specific Cox Models Depicting the Possibility of All-Cause Mortality, MACE, ESKD, and Readmission **eTable 11.** Cause-Specific Cox Models Depicting the Possibility of All-Cause Mortality, MACE, ESKD, and Readmission With Discharge Date as the Time Zero Point **eTable 12.** Specificity Analysis for Risk of 3 Independent Events, Appendicitis, Traffic Accident, and Deafness Among Different Baseline CKD Stages **eTable 13.** Specificity Analysis for Risk of 3 Independent Events, Appendicitis, Traffic Accident, and Deafness Among Different AKD Stages **eTable 14.** Specificity Analysis for Risk of 3 Independent Events, Appendicitis, Traffic Accident, and Deafness Among Different Post-AKI CKD Stages

Supplement 2.
Data Sharing Statement
