# Supplementary material for: Recovery Dynamics and Prognosis After Dialysis for Acute Kidney Injury
Source: JAMA Netw Open. 2024 Mar 8;7(3):e240351. doi: 10.1001/jamanetworkopen.2024.0351 (PMC10924241; doi:10.1001/jamanetworkopen.2024.0351)
Supplement: Supplement 1. — eAppendix. Supplementary Material and Methods eFigure 1. Time Course of the Study eFigure 2. The Risk for the Adverse Outcomes in the Stages of AKD, Baseline CKD, and Post-AKD CKD eFigure 3. Sankey Diagrams Showing the Natural Course of Kidney Function in the Patients Who Had an AKI Episode eFigure 4. Forest Plots of Subgroup Analyses According to Baseline Characteristics for Stage 3-5 vs. Stage 0-2 After AKD eFigure 5. The Influence of Overlap Weighting on Covariate Balance and Treatment Effect Estimation eFigure 6. Graphic Abstract eTable 1. Diagnosis Codes Used in the Study eTable 2. Medication Codes Used in This Study eTable 3. Baseline Characteristics of Enrolled Patients and Excluded Patients eTable 4. Cox Proportional Hazards Models for Adverse Outcomes, Fitting Models With Respect to Baseline Kidney Function, AKD Severity, and Post-AKD Kidney Function (Separate Models for Each Exposure) eTable 5. Correlation Between Baseline Kidney Function, AKD Severity, and Post-AKD Kidney Function eTable 6. Patient Distribution According to the Combination of Baseline Kidney Function and Post-AKD Kidney Function eTable 7. Sensitivity Analysis for Risk of Mortality Among Different Post-AKI-CKD Stages eTable 8. Sensitivity Analysis for Risk of MACE or Mortality Among Different Post-AKI-CKD Stages eTable 9. Sensitivity Analysis for Risk of All-Cause Mortality, MACE, ESKD, and Readmission Across Different Post-AKI-CKD Stages With Discharge Date as the Time Zero Point eTable 10. Cause-Specific Cox Models Depicting the Possibility of All-Cause Mortality, MACE, ESKD, and Readmission eTable 11. Cause-Specific Cox Models Depicting the Possibility of All-Cause Mortality, MACE, ESKD, and Readmission With Discharge Date as the Time Zero Point eTable 12. Specificity Analysis for Risk of 3 Independent Events, Appendicitis, Traffic Accident, and Deafness Among Different Baseline CKD Stages eTable 13. Specificity Analysis for Risk of 3 Independent Events, Appendicitis, Traffic Accident, [file jamanetwopen-e240351-s001.pdf]

Supplementary Online Content

Pan HC, Chen HY, Teng NC, et al. Baseline kidney function and recovery dynamics after long-term dialysis. *JAMA Netw Open*. 2024;7(3):e240351. doi:10.1001/jamanetworkopen.2024.0351

eAppendix. Supplementary Material and Methods

eFigure 1. Time Course of the Study

eFigure 2. The Risk for the Adverse Outcomes in the Stages of AKD, Baseline CKD, and Post-AKD CKD

eFigure 3. Sankey Diagrams Showing the Natural Course of Kidney Function in the Patients Who Had an AKI Episode

eFigure 4. Forest Plots of Subgroup Analyses According to Baseline Characteristics for Stage 3-5 vs. Stage 0-2 After AKD

eFigure 5. The Influence of Overlap Weighting on Covariate Balance and Treatment Effect Estimation

eFigure 6. Graphic Abstract

eTable 1. Diagnosis Codes Used in the Study

eTable 2. Medication Codes Used in This Study

eTable 3. Baseline Characteristics of Enrolled Patients and Excluded Patients

eTable 4. Cox Proportional Hazards Models for Adverse Outcomes, Fitting Models With Respect to Baseline Kidney Function, AKD Severity, and Post-AKD Kidney Function (Separate Models for Each Exposure)

eTable 5. Correlation Between Baseline Kidney Function, AKD Severity, and Post-AKD Kidney Function

eTable 6. Patient Distribution According to the Combination of Baseline Kidney Function and Post-AKD Kidney Function

eTable 7. Sensitivity Analysis for Risk of Mortality Among Different Post-AKI-CKD Stages

eTable 8. Sensitivity Analysis for Risk of MACE or Mortality Among Different Post-AKI-CKD Stages

eTable 9. Sensitivity Analysis for Risk of All-Cause Mortality, MACE, ESKD, and Readmission Across Different Post-AKI-CKD Stages With Discharge Date as the Time Zero Point

eTable 10. Cause-Specific Cox Models Depicting the Possibility of All-Cause Mortality, MACE, ESKD, and Readmission

eTable 11. Cause-Specific Cox Models Depicting the Possibility of All-Cause Mortality, MACE, ESKD, and Readmission With Discharge Date as the

© 2024 Pan HC et al. *JAMA Network Open.*

Time Zero Point
eTable 12. Specificity Analysis for Risk of 3 Independent Events, Appendicitis, Traffic Accident, and Deafness Among Different Baseline CKD Stages
eTable 13. Specificity Analysis for Risk of 3 Independent Events, Appendicitis, Traffic Accident, and Deafness Among Different AKD Stages
eTable 14. Specificity Analysis for Risk of 3 Independent Events, Appendicitis, Traffic Accident, and Deafness Among Different Post-AKI CKD Stages

This supplementary material has been provided by the authors to give readers additional information about their work.

© 2024 Pan HC et al. *JAMA Network Open.*

eAppendix. Supplementary Material and Methods

Introduction to the NHIRD

The National Health Insurance Research Database (NHIRD) is a comprehensive clinical database in Taiwan. Established by Taiwan's National Health Insurance (NHI), a single-payer system initiated by the government in 1995, it now encompasses the medical records of over 99% of Taiwan's 23 million residents. This database meticulously captures details on outpatient visits, inpatient stays, emergency interventions, and various medical and surgical procedures. Additionally, it offers granular personal details such as gender, date of birth, insurance tier, and residency, all available for research endeavors. The depth of clinical information and its extensive reach predominantly influenced our decision to adopt NHIRD for this study. Moreover, the Applied Health Research Data Integration Service offers a conduit for researchers to interlink laboratory databases with claims records[1].

eReference

1. Lee P-C, Kao F-Y, Liang F-W, Lee Y-C, Li S-T, Lu T-H. Existing data sources in clinical epidemiology: The Taiwan national health insurance laboratory databases. *Clinical Epidemiology.* 2021:175-181.

© 2024 Pan HC et al. *JAMA Network Open.*

Supplementary material and methods: Propensity scores for multiple treatments

In our study, we used the 'twang' package in R for propensity score weighting. This package is designed for non-equivalent groups and facilitates the simultaneous analysis of multiple treatment groups. It utilizes a tree-based generalized boosted regression model to calculate propensity scores and the corresponding weights for estimating the population's average treatment effect (ATE). The propensity scores were computed using the 'mnps' function within the 'twang' package[1,2], which employs boosted logistic regression to determine the likelihood of an individual belonging to one of the treatment groups. It operates by repeatedly applying the "ps" function to compare each treatment group against a combined sample of the other groups. The key outcome of this process is the ATE, which measures the impact if all individuals in the target population were assigned to one specific treatment group versus another. MNPS aims to achieve balance in baseline variables that show significant differences across treatment groups, generating appropriate propensity weights for each case. This leads to balanced distributions of baseline variables across groups[3,4]. The clinical factors entered into the propensity score weighting were including demographics (age, sex), health conditions (CCI score, baseline kidney function, AKD severity, hypertension, diabetes, hyperuricemia, congestive heart failure, cerebrovascular diseases, chronic obstructive pulmonary disease, malignancy), Intervention during index hospitalization (Hospitalization days, ICU admission, MV, PMV, ARDS, CABG, PTCA, IABP, ECMO, major operation), AKI contributors (sepsis, hypovolemic shock, CT with contrast, Other or mixed causes), medications (Antiplatelet, Statin, Urate-lowering drug, alpha-blocker, beta-blocker, calcium channel blocker, ACEI or ARB, MRA, other anti-hypertensives), and clinical metrics after discharge (BUN and eGFR). To validate the efficacy of our propensity models, we conducted a graphical analysis, examining the absolute standardized mean differences in covariates between the treatment groups. The demographic characteristics of the groups, adjusted by propensity weighting, were then compared using t-tests and chi-squared tests.

Abbreviations: ACEI, angiotensin converting enzyme inhibitors**;** AKD, acute kidney disease; AKI, acute kidney injury; ANOVA, Analysis of Variance; ARB, angiotensin receptor blockers*;* ARDS, acute respiratory distress syndrome**;** CABG, coronary artery bypass graft; CCB, calcium *channel* blocker**;** CCI, Charlson Comorbidity Index; CKD, chronic kidney disease; COPD, chronic obstructive pulmonary disease; CT, computerized tomography; ECMO, extra-corporeal membrane oxygenation**;** IABP, intra-aortic balloon pump**;** ICU, intensive care unit; MACE, major adverse cardiac

© 2024 Pan HC et al. *JAMA Network Open.*

event; MRA, mineralocorticoid receptor antagonists; MV, mechanical ventilation; PMV, persistent mechanical ventilation; PTCA, percutaneous transluminal coronary angioplasty.

eReferences

1. Burgette L, Griffin BA, McCaffrey D. Propensity scores for multiple treatments: A tutorial for the mnps function in the twang package. *R package. Rand Corporation.* 2017.
2. Cefalu M, McCaffrey D, Morral A, Griffin B, Burgette L. Toolkit for weighting and analysis of nonequivalent groups. 2021.
3. Schmidt C, Borgia M, Zhang T, Gochyyev P, Shireman TI, Resnik L: **Initial treatment approaches and healthcare utilization among veterans with low back pain: a propensity score analysis**. *BMC Health Serv Res* 2023, **23**(1):275.
4. Palacios J, Adegoke A, Wogan R, Duffy D, Earley C, Eilert N, Enrique A, Sollesse S, Chapman J, Richards D: **Comparison of outcomes across low-intensity psychological interventions for depression and anxiety within a stepped-care setting: A naturalistic cohort study using propensity score modelling**. *Br J Psychol* 2023, **114**(2):299-314.

© 2024 Pan HC et al. *JAMA Network Open.*

eFigure 1. Timing to assess kidney function of this study

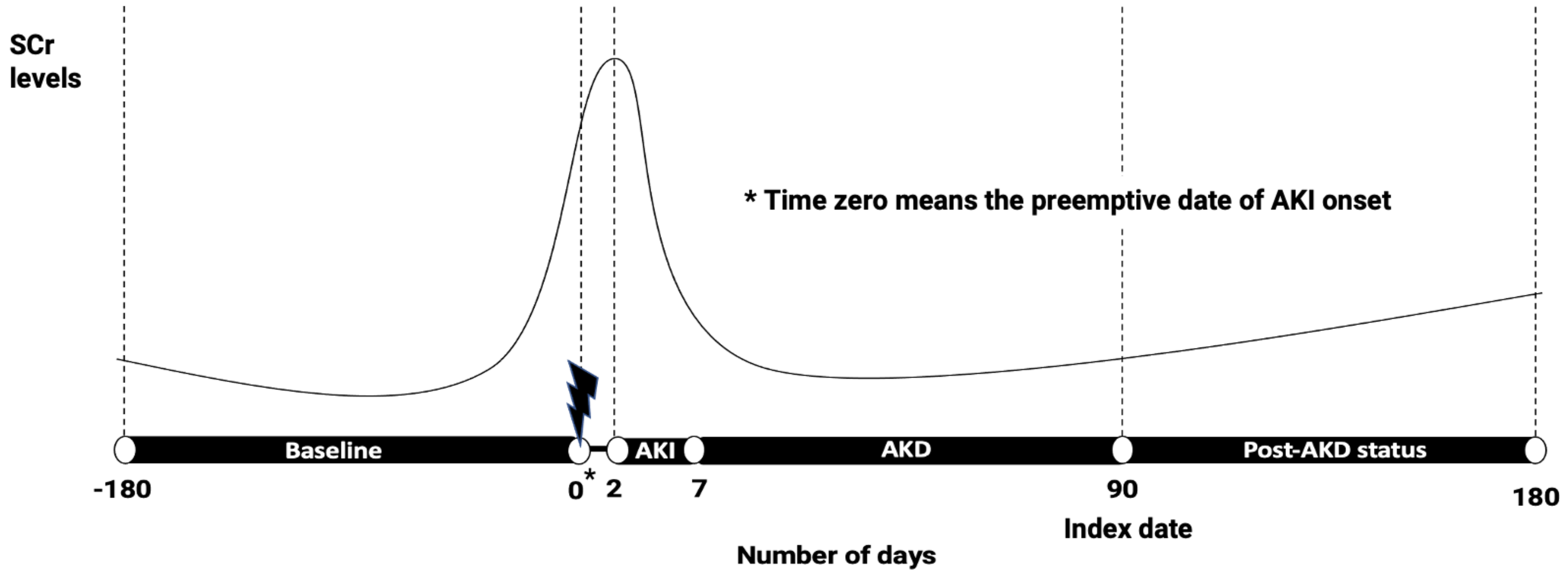

Abbreviations: AKD, acute kidney disease; AKI, acute kidney injury.

© 2024 Pan HC et al. *JAMA Network Open.*

eFigure 2. The risks for the adverse outcomes in the stages of AKD, baseline CKD, and post-AKD CKD. The height of each bar represents the HR ot sHR for the adverse events in the stages of AKD, baseline CKD, and post-AKD CKD (Reference: Baseline CKD stage 0-2, AKD stage 0, and post-AKD CKD stage 0-2)

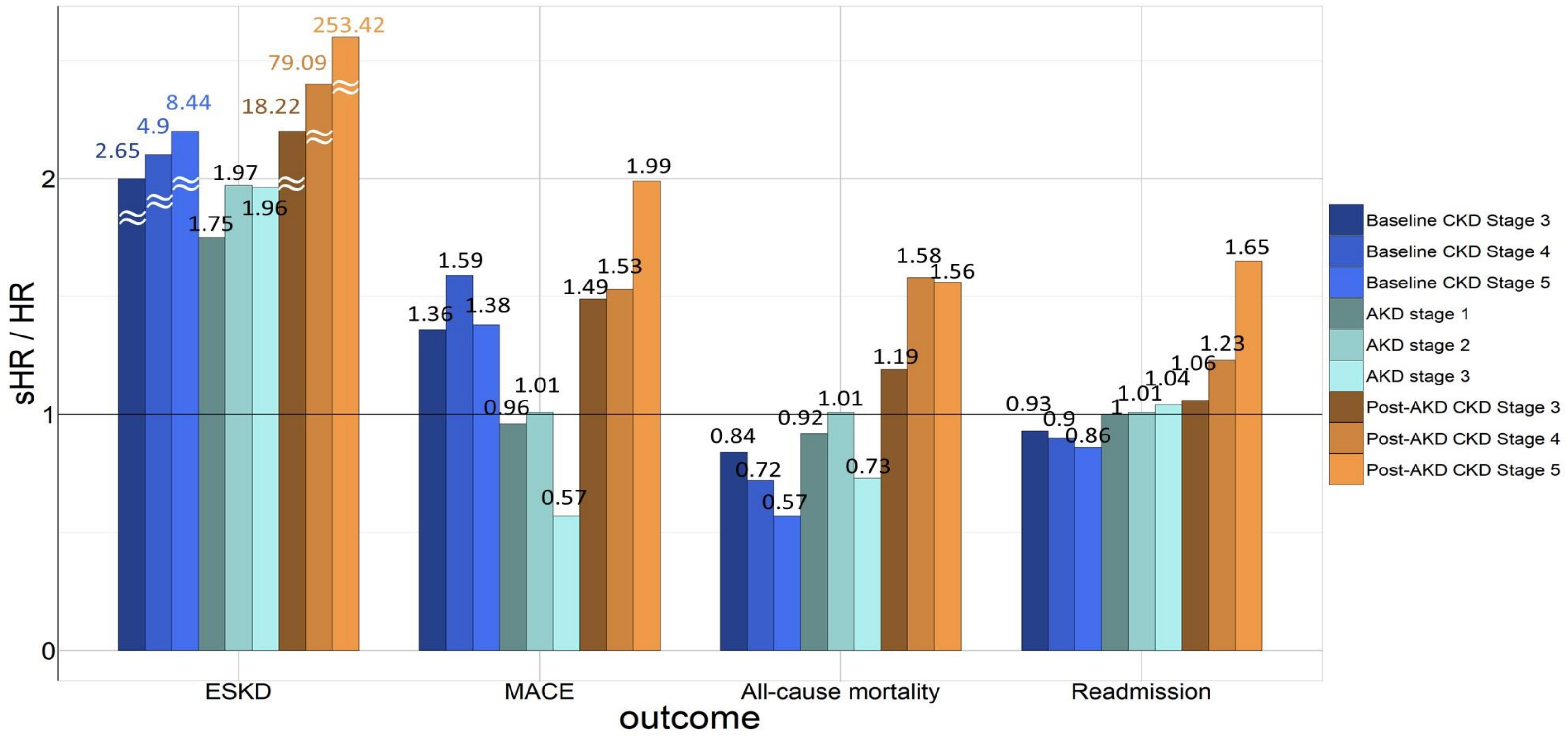

Abbreviations: AKD, acute kidney disease; CKD, chronic kidney disease; ESKD, end-stage kidney disease; HR, hazard ratio; MACE, major adverse cardiac events; sHR, Sub-distribution hazard ratio

© 2024 Pan HC et al. *JAMA Network Open.*

eFigure 3. Sankey diagrams showing the natural course of kidney function in the patients who had an AKI episode. (A) Baseline CKD - AKD – clinical kidney outcome (B) Baseline CKD - post-AKD CKD – clinical kidney outcome.

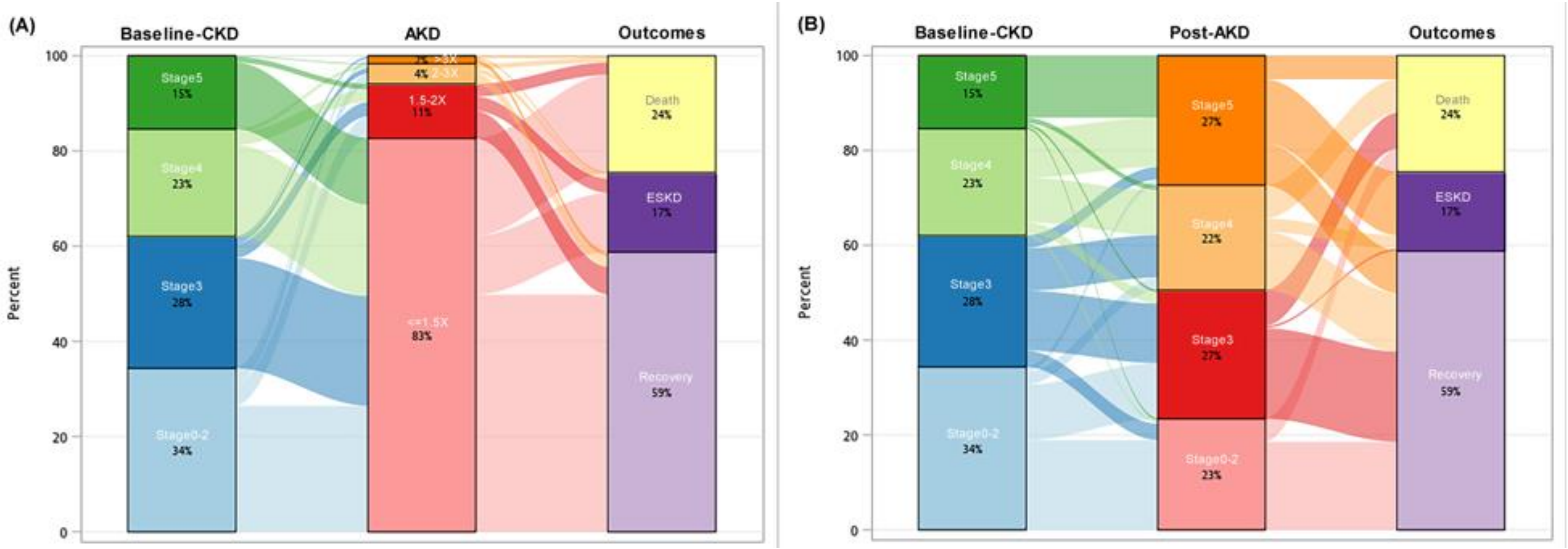

Abbreviations: AKD, acute kidney disease; AKI, acute kidney injury; CKD, chronic kidney disease; ESKD, end-stage kidney disease; RRT, renal replacement therapy; SCr, serum creatinine

© 2024 Pan HC et al. *JAMA Network Open.*

eFigure 4. Forest plots of subgroup analyses according to baseline characteristics for stage 3-5 vs. stage 0-2 after AKD

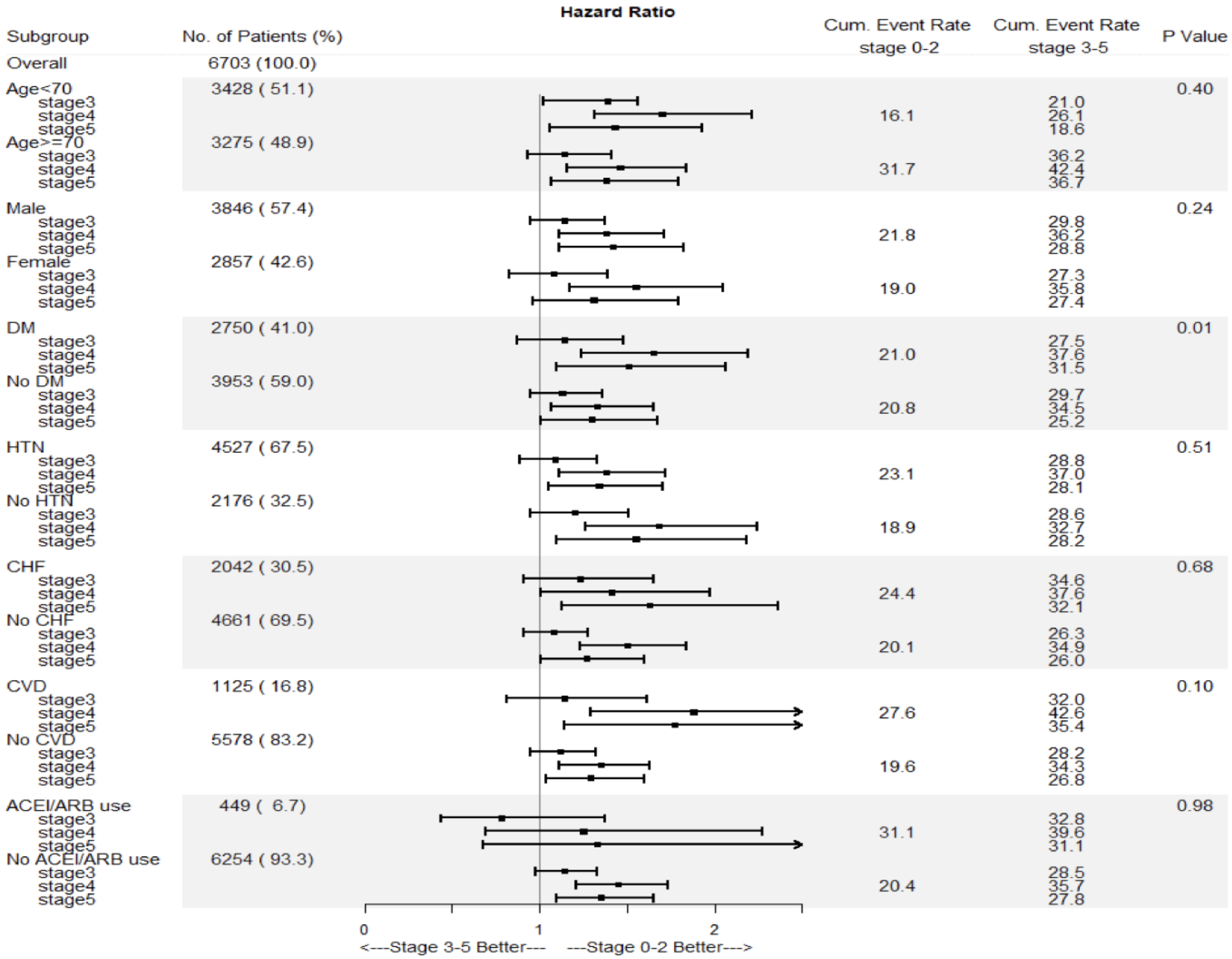

Abbreviations: ACEI, angiotensin-converting enzyme inhibitor; AKD, acute kidney disease; ARB, angiotensin receptor blocker; CHF, congestive heart failure; CVD, cardiovascular disease; DM, diabetes mellitus; HTN, hypertension

© 2024 Pan HC et al. *JAMA Network Open.*

eFigure 5. The influence of overlap weighting on covariate balance and treatment effect estimation. After weighting, the maximum absolute standardized mean differences decrease for all pretreatment covariates. The statistically significant difference (before taking the maximum across treatment groups) is indicated by the solid circle.

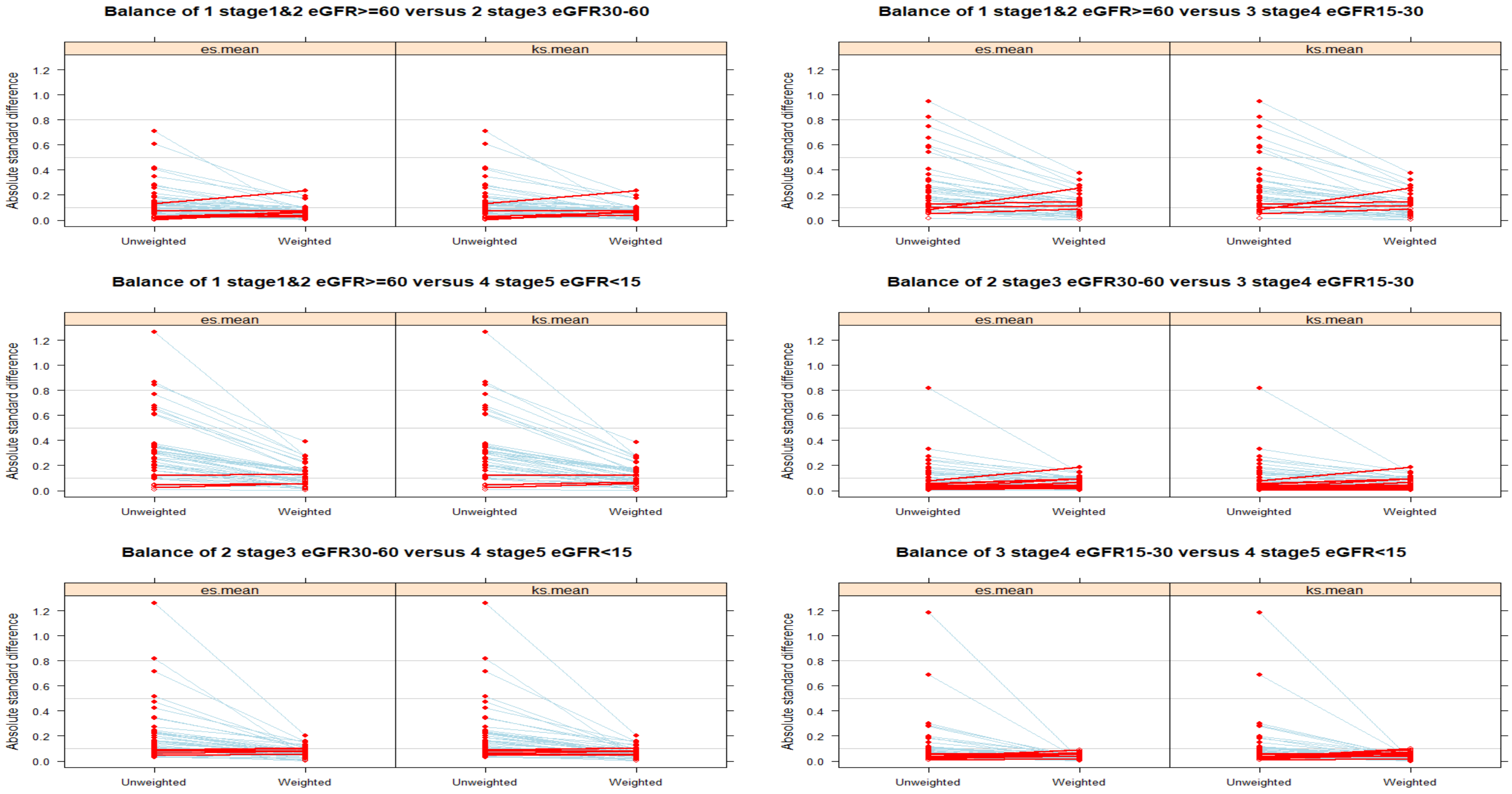

Abbreviations: eGFR, estimated glomerular filtrate rate

© 2024 Pan HC et al. *JAMA Network Open.*

eFigure 6. Graphic abstract

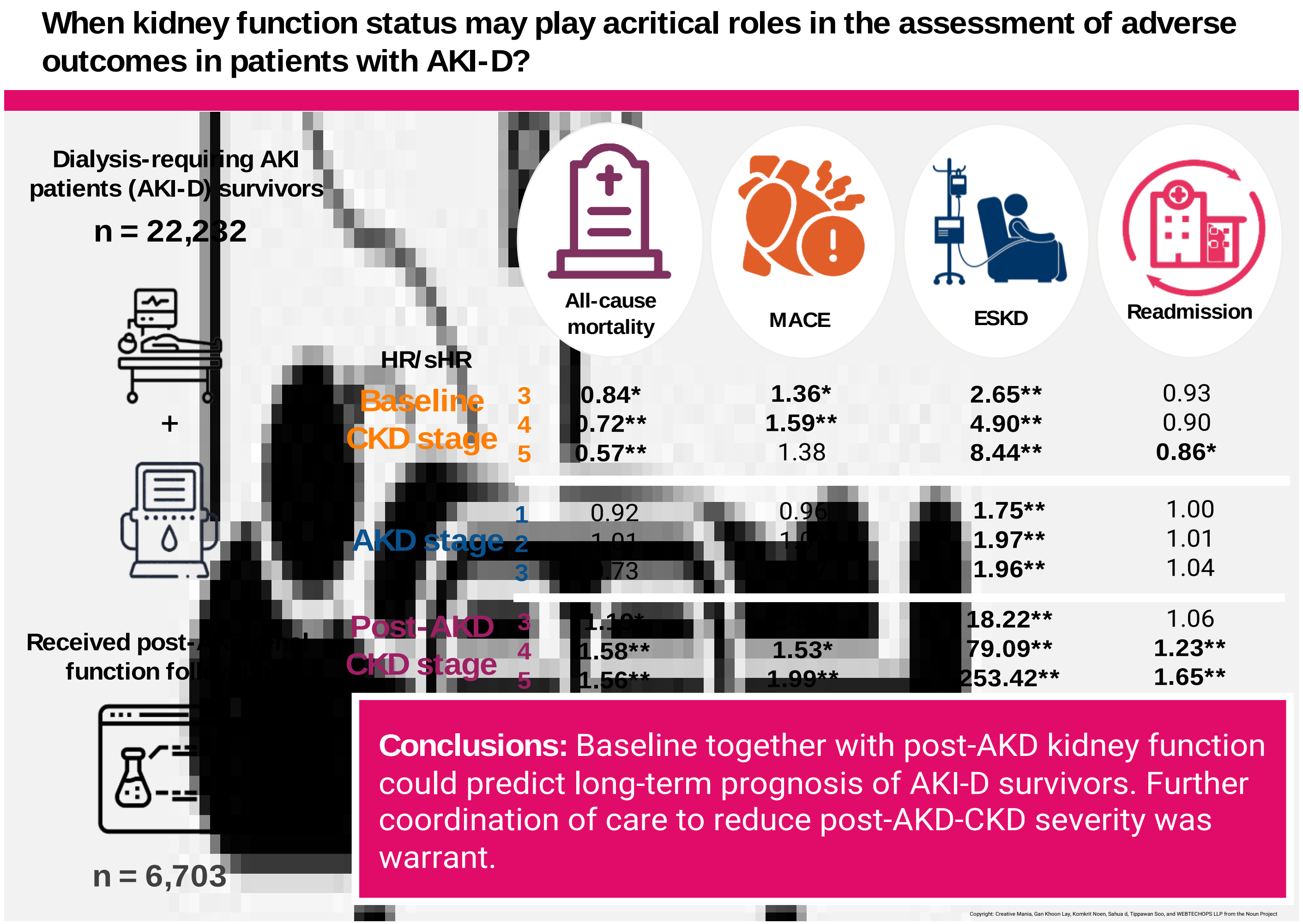

Abbreviations: AKD, acute kidney disease; AKI-D, dialysis-requiring acute kidney injury; CKD, chronic kidney disease; ESKD, end-stage kidney disease; HR, hazard ratio; MACE, major adverse cardiac event

* P < 0.05

** P < 0.01

© 2024 Pan HC et al. *JAMA Network Open.*

eTable 1. Diagnosis codes used in the study

Disease	ICD-9-CM codes and ICD-10-CM
Diabetes mellitus	250, 250.0, 250.1, 250.2, 250.3, 250.7, 250.00, 250.01, 250.10, 250.11, 250.20, 250.21, 250.30, 250.31, 250.70, 250.71, E10.1, E10.5, E10.6, E10.9, E11.0, E11.5, E11.6, E11.9
Hypertension	401, 401.0, 401.1, 401.9, 402, 402.0, 402.00, 402.01, 402.1, 402.10, 402.11, 402.9, 402.90, 402.91, 403, 403.0, 403.00, 403.01, 403.1, 403.10, 403.11, 403.9, 403.90, 403.91, 404, 404.0, 404.00, 404.01, 404.02, 404.03, 404.1, 404.10, 404.11, 404.12, 404.13, 404.9, 404.90, 404.91, 404.92, 404.93, 405, 405.0, 405.01, 405.09, 405.1, 405.11, 405.19, 405.9, 405.91, 405.99, I11.0, I11.9, I12.0, I12.9, I13.0, I13.11, I13.2, I15.0, I15.8, I15.1, N26.2, I15.2, I15.9, I10, I13.10
Acute respiratory distress syndrome	518.5, 518.81, 518.82, J95.1, J95.2, J95.3, J95.82, J96.0, J96.9, J80, Z87.09
Pleural effusion	511.1, 511.9, 511.0, J90, J94.8, J91.8, J92.0, J92.9, J94.1, J94.9, R09.1
Hypovolemic shock	785.59, R57.1
Congestive Heart Failure	398.91, 402.01, 402.11, 402.91, 404.01, 404.03, 404.11, 404.13, 404.91, 404.93, 425.4, 425.5, 425.7, 425.8, 425.9, 428, I43, I50, I09.9, I11.0, I13.0, I13.2, I25.5, I42.0, I42.5, I42.6, I42.7, I42.8, I42.9, P29.0
Hyperlipidemia	272, 272.1, 272.2, 272.3, 272.4, 272.5, 272.6, 272.7, E78.0, E78.1, E78.2, E78.3, E78.4, E78.5, E78.6, E88.1, E75.21, E75.22, E75.240, E75.241, E75.242, E75.243, E75.248, E75.249, E75.3, E77.0, E77.1, E77.8
Hyperuricemia	274, M10
Cerebrovascular Disease	362.34, 430, 431, 432, 433, 434, 435, 436, 437, 438, G45, G46, I60, I61, I62, I63, I64, I65, I66, I67, I68, I69, H34.0
Malignancies	140, 141, 142, 143, 144, 145, 146, 147, 148, 149, 150, 151, 152, 153, 154, 155, 156, 157, 158, 159, 160, 161, 162, 163, 164, 165, 170, 171, 172, 174, 175, 176, 179, 180, 181, 182, 183, 184, 185, 186, 187, 188, 189, 190, 191, 192, 193, 194, 195, 200, 201, 202, 203, 204, 205, 206, 207, 208, 238.6, C00, C01, C02, C03, C04, C05, C06, C07, C08, C09, C10, C11, C12, C13, C14, C15, C16, C17, C18, C19, C20, C21, C22, C23, C24, C25, C26, C30, C31, C32, C33, C34, C37, C38, C39, C40, C41, C43, C45, C46, C47, C48, C49, C50, C51, C52, C53, C54, C55, C56, C57, C58, C60, C61, C62,

© 2024 Pan HC et al. *JAMA Network Open.*

	C63, C64, C65, C66, C67, C68, C69, C70, C71, C72, C73, C74, C75, C76, C81, C82, C83, C84, C85, C88, C90, C91, C92, C93, C94, C95, C96, C97
Myocardial Infarction	410, 412, I21, I22, I25.2
Chronic Lung Disease	416.8, 416.9, 490, 491, 492, 493, 494, 495, 496, 500, 501, 502, 503, 504, 505, 506.4, 508.1, 508.8, J40, J41, J42, J43, J44, J45, J46, J47, J60, J61, J62, J63, J64, J65, J66, J67, I27.8, I27.9, J68.4, J70.1, J70.3
Sepsis (Infectious disease + Organ dysfunction)	Infectious disease<br>001, 002, 003, 004, 005, 008, 009, 010, 011, 012, 013, 014, 015, 016, 017, 018, 020, 021, 022, 023, 024, 025, 026, 027, 030, 031, 032, 033, 034, 035, 036, 037, 038, 039, 040, 041, 090, 091, 092, 093, 094, 095, 096, 097, 098, 100, 101, 102, 103, 104, 110, 111, 112, 114, 115, 116, 117, 118, 320, 322, 324, 325, 420, 421, 451, 461, 462, 463, 464, 465, 481, 482, 485, 486, 494, 510, 513, 540, 541, 542, 566, 567, 590, 597, 601, 614, 615, 616, 681, 682, 683, 686, 730, 5695, 5720, 5721, 5750, 5990, 7110, 7907, 9966, 9985, 9993, 49121, 56201, 56203, 56211, 56213, 56983, A000, A001, A009, A0100, A0101, A0102, A0103, A0104, A0105, A0109, A011, A012, A013, A014, A020, A021, A0220, A0221, A0222, A0223, A0224, A0225, A0229, A028, A029, A030, A031, A032, A033, A038, A039, A050, A051, A052, A058, A053, A055, A054, A059, A044, A040, A041, A042, A043, A048, A045, A046, A047, A049, A080, A082, A0811, A0819, A0831, A0832, A0839, A084, A088, A09, A157, A156, A150, A155, A154, A158, A159, A170, A171, A1781, A1782, A1783, A1789, A179, A1831, A1832, A1839, A1883, A1801, A1802, A1803, A1809, A1811, A1812, A1813, A1815, A1814, A1817, A1816, A1818, A1810, A184, A182, A1850, A1851, A1852, A1853, A1854, A1859, A186, A1881, A187, A1885, A1889, A1882, A1884, A190, A191, A192, A198, A199, A200, A201, A207, A202, A203, A208, A209, A210, A213, A212, A211, A217, A218, A219, A220, A221, A222, A227, A228, A229, A230, A231, A232, A233, A238, A239, A240, A241, A242, A243, A249, A250, A251, A259, A320, A3211, A3212, A327, A3281, A3282, A3289, A329, A260, A267, A268, A269, A280, A282, A288, A289, A305, A301, A300, A302, A303, A304, A308, A309, A310, A311, A312, A318, A319, A360, A361, A3689, A362, A3686, A3681, A3685, A363, A3682, A3683, A3684, A369, A3700, A3710, A3780, A3790, J020, J0300, J0301, A380, A381, A388, A389, A46, A390, A3981, A392, A393, A394, A391, A3950, A3953, A3951, A3952, A3982, A3983, A3984, A3989, A399, A35,

© 2024 Pan HC et al. *JAMA Network Open.*

	A400, A401, A408, A409, R6510, R6511, R6520, A412, A4101, A4102, A411, A403, A414, A4150, A413, A4151, A4152, A4153, A4159, A4181, A4189, A419, A431, L081, A420, A430, A421, A422, B479, A4281, A4282, A4289, A438, A429, A439, B471, A480, A488, K9081, M60009, A482, A483, A484, A491, B955, B950, B951, B954, B952, B953, B958, A4901, A4902, B9561, B9562, B957, B961, B9620, B9621, B9622, B9623, B9629, A492, B963, A498, B964, B965, A493, B960, B966, B967, B9682, B9689, B9681, A499, A5001, A5002, A5003, A5004, A5005, A5006, A5007, A5008, A5009, A501, A502, A5031, A5040, A5043, A5045, A5042, A5041, A5049, A5030, A5032, A5039, A5044, A5051, A5052, A5053, A5054, A5055, A5056, A5057, A5059, A506, A507, A509, A510, A511, A512, A5131, A5139, A5149, A5143, A5146, A5145, A5141, A5132, A5142, A5144, A515, A519, A5201, A5202, A5203, A5206, A5204, A5205, A5209, A5200, A5211, A5217, A5213, A522, A5214, A5219, A5215, A5212, A5210, A523, A5271, A5272, A5274, A5275, A5277, A5278, A5273, A5276, A5279, A528, A529, A530, A539, A5400, A5402, A5409, A541, A5429, A5401, A5422, A5423, A5403, A5424, A5421, A5431, A5432, A5439, A5433, A5430, A5442, A5449, A5441, A5440, A5443, A545, A546, A5489, A5481, A5483, A5485, A5482, A5484, A5486, A549, A270, A2781, A2789, A279, A690, A691, A660, A661, A662, A663, A664, A665, A666, A667, A668, A669, A670, A671, A672, A673, A679, A65, A698, A699, B350, B351, B352, B356, B353, B354, B355, B358, B359, B360, B361, B362, B363, B368, B369, B370, B3783, B373, B3741, B3742, B3749, B372, B371, B377, B376, B3784, B375, B3781, B3782, B3789, B379, B380, B383, B3881, B384, B387, B3889, B381, B382, B389, B394, G02, H32, I32, I39, B390, B391, B392, B393, B395, J17, B399, B400, B401, B402, B403, B407, B4081, B4089, B409, B410, B417, B418, B419, B480, B481, B420, B421, B427, B4281, B4282, B4289, B429, B430, B431, B432, B438, B439, B441, B442, B447, B4489, B449, B484, B470, B450, B452, B453, B457, B458, B459, B482, B460, B461, B462, B463, B464, B465, B468, B469, B488, B483, B49, G000, G001, G002, G003, G01, G008, G009, G042, G030, G038, G031, G039, G060, G061, G062, G07, G08, I301, I309, I300, I308, I330, I339, I8000, I8001, I8002, I8003, I8010, I8011, I8012, I8013, I80201, I80202, I80203, I80209, I80221, I80222, I80223, I80229, I80231, I80232, I80233, I80239, I80291, I80292, I80293, I80299, I803, I80211, I80212, I80213, I80219, I808, I809, J0100, J0101, J0110, J0111, J0120, J0121, J0130, J0131, J0140, J0141, J0180, J0181, J0190, J0191, J028, J029, J0380, J0381, J0390, J0391, J040, J0430, J0431, J050,

© 2024 Pan HC et al. *JAMA Network Open.*

	J0410, J0411, J042, J0510, J0511, J060, J069, J13, J181, J150, J151, J14, J154, J153, J1520, J15211, J1529, J158, J155, J156, A481, J159, J180, J188, J189, J440, J441, J479, J470, J471, J860, J869, J850, J851, J852, J853, K352, K353, K3580, K3589, K37, K36, K5700, K5712, K5701, K5713, K5720, K5732, K5740, K5752, K5792, K5721, K5733, K5781, K5793, K610, K611, K612, K613, K614, K67, K658, K650, K651, K652, K6811, K6819, K653, K654, K659, K689, K630, K631, K750, K751, K810, N110, N118, N10, N151, N2884, N2885, N2886, N119, N12, N136, N16, N159, N340, N341, N342, N343, N390, N410, N411, N412, N413, N51, N414, N418, N419, N7001, N7002, N7003, N7011, N7012, N7013, N7091, N7092, N7093, N730, N731, N732, N733, N736, N734, N738, N74, N735, N739, N710, N711, N719, N72, N760, N761, N762, N763, N94810, N771, N750, N758, N751, N764, N766, N770, N759, N765, N7681, N7689, L02511, L02512, L02519, L03011, L03012, L03019, L03021, L03022, L03029, L02611, L02612, L02619, L03031, L03032, L03039, L03041, L03042, L03049, K122, L0201, L03211, L03212, L0211, L03221, L03222, L02211, L02212, L02213, L02214, L02215, L02216, L02219, L03311, L03312, L03313, L03314, L03315, L03316, L03319, L03321, L03322, L03323, L03324, L03325, L03326, L03329, L02411, L02412, L02413, L02414, L02419, L03111, L03112, L03113, L03114, L03119, L03121, L03122, L03123, L03124, L03129, L0231, L03317, L03327, L02415, L02416, L03115, L03116, L03125, L03126, L02811, L02818, L03811, L03818, L03891, L03898, L0291, L0390, L0391, L983, L040, L041, L042, L043, L048, L049, L080, L88, L0881, L0889, L928, L980, B781, E832, L0882, L089, M0000, M0010, M0020, M0080, M009, M00011, M00012, M00019, M00111, M00112, M00119, M00211, M00212, M00219, M00811, M00812, M00819, M00021, M00022, M00029, M00121, M00122, M00129, M00221, M00222, M00229, M00821, M00822, M00829, M00031, M00032, M00039, M00131, M00132, M00139, M00231, M00232, M00239, M00831, M00832, M00839, M00041, M00042, M00049, M00141, M00142, M00149, M00241, M00242, M00249, M00841, M00842, M00849, M00051, M00052, M00059, M00151, M00152, M00159, M00251, M00252, M00259, M00851, M00852, M00859, M00061, M00062, M00069, M00161, M00162, M00169, M00261, M00262, M00269, M00861, M00862, M00869, M00071, M00072, M00079, M00171, M00172, M00179, M00271, M00272, M00279, M00871, M00872, M00879, M0008, M0018, M0028, M0088, M0009, M0019, M0029, M0089, M8600, M8610, M8620, M86011, M86012, M86019, M86111, M86112, M86119, M86211, M86212,

© 2024 Pan HC et al. *JAMA Network Open.*

	M86219, M86021, M86022, M86029, M86121, M86122, M86129, M86221, M86222, M86229, M86031, M86032, M86039, M86131, M86132, M86139, M86231, M86232, M86239, M86041, M86042, M86049, M86141, M86142, M86149, M86241, M86242, M86249, M86051, M86052, M86059, M86151, M86152, M86159, M86251, M86252, M86259, M86061, M86062, M86069, M86161, M86162, M86169, M86261, M86262, M86269, M86071, M86072, M86079, M86171, M86172, M86179, M86271, M86272, M86279, M8608, M8618, M8628, M8609, M8619, M8629, M8630, M8640, M8650, M8660, M868X9, M86311, M86312, M86319, M86411, M86412, M86419, M86511, M86512, M86519, M86611, M86612, M86619, M868X1, M86321, M86322, M86329, M86421, M86422, M86429, M86521, M86522, M86529, M86621, M86622, M86629, M868X2, M86331, M86332, M86339, M86431, M86432, M86439, M86531, M86532, M86539, M86631, M86632, M86639, M868X3, M86341, M86342, M86349, M86441, M86442, M86449, M86541, M86542, M86549, M86641, M86642, M86649, M868X4, M86351, M86352, M86359, M86451, M86452, M86459, M86551, M86552, M86559, M86651, M86652, M86659, M868X5, M86361, M86362, M86369, M86461, M86462, M86469, M86561, M86562, M86569, M86661, M86662, M86669, M868X6, M86371, M86372, M86379, M86471, M86472, M86479, M86571, M86572, M86579, M86671, M86672, M86679, M868X7, M8638, M8648, M8658, M8668, M868X8, M8639, M8649, M8659, M8669, M868X0, M869, M4620, M4621, M4622, M4623, M4624, M4625, M4626, M4627, M4628, M8960, M89611, M89612, M89619, M89621, M89622, M89629, M89631, M89632, M89639, M89641, M89642, M89649, M89651, M89652, M89659, M89661, M89662, M89669, M89671, M89672, M89679, M8968, M8969, M9080, M90811, M90812, M90819, M90821, M90822, M90829, M90831, M90832, M90839, M90841, M90842, M90849, M90851, M90852, M90859, M90861, M90862, M90869, M90871, M90872, M90879, M9088, M9089, M4630, M4631, M4632, M4633, M4634, M4635, M4636, M4637, M4638, M4639, R7881, T8579XA, T826XXA, T827XXA, T80211A, T80212A, T80218A, T80219A, T8022XA, T8351XA, T8359XA, T836XXA, T8450XA, T8451XA, T8452XA, T8453XA, T8454XA, T8459XA, T8460XA, T84610A, T84611A, T84612A, T84613A, T84614A, T84615A, T84619A, T84620A, T84621A, T84622A, T84623A, T84624A, T84625A, T84629A, T8463XA, T8469XA, T847XXA, T8571XA, T8572XA, T86842, T814XXA, R5084, N980, T8029XA, T880XXA

© 2024 Pan HC et al. *JAMA Network Open.*

	Organ dysfunction
	4580, 4588, 4589, 7855, 78551, 78559, 7963, 51881, 51882, 51885, 78609, 7991, 967, 9671, 9672, 9604, 9390, 580, 5800, 5804, 5808, 58081, 58089, 5809, 584, 5845, 5846, 5847, 5848, 5849, 586, 3995, 570, 5722, 5733, 5734, 293, 3481, 3483, 78001, 78009, 8914, 2862, 2866, 2869, 2873, 2874, 2875, 79092, 2762, I951, I9589, I959, R579, R570, H49811, H49812, H49813, R571, R578, R6521, R031, J9600, J9601, J9602, J9690, J9691, J9692, J80, R0600, R0609, R063, R0683, R0689, R092, T423X1A, T423X2A, T423X3A, T423X4A, T426X1A, T364X1A, T364X2A, T364X3A, T364X4A, T190XXA, T191XXA, N000, N001, N002, N003, N004, N005, N006, N007, N010, N011, N012, N013, N014, N015, N016, N017, N018, N019, N08, N008, N009, N170, N171, N172, N178, N179, N19, K7200, K7201, K761, K762, K7290, K7291, K710, K7110, K7111, K712, K713, K714, K7150, K7151, K716, K717, K718, K719, K752, K753, K7581, K7589, K759, K764, K763, F05, F062, F060, F0630, F0631, F0632, F0633, F0634, F064, F061, F53, F068, G931, G9340, G9341, G9349, I6783, E035, R4020', R402110, R402111, R402112, R402113, R402114, R402120, R402121, R402122, R402123, R402124, R402210, R402211, R402212, R402213, R402214, R402220, R402221, R402222, R402223, R402224, R402310, R402311, R402312, R402313, R402314, R402320, R402321, R402322, R402323, R402324, R402340, R402341, R402342, R402343, R402344, R400, R401, R410, D681, D65, D688, D689, D473, D693, D6941, D6942, D6949, D6951, D6959, D7582, D696, R791, E872
Appendicitis	540.0, 540.1, 540.9, 540.9, 541, 542, 543.0, 543.9, K35, K35.2, K35.3, K35.80, K35.89, K37, K36, K38.0, K38.1, K38.2, K38.3, K38.8, K38.9, E800-E844, V00-V99
Traffic accident	E800-E844, V00-V99
Deafness	389, 389.01, 389.02, 389.03, 389.04, 389.08, 389.1, 389.11, 389.12, 389.14, 389.18, 389.2, 389.7, 389.8, 389.9, H90.0, H90.11, H90.12, H90.2, H90.5, H90.3, H90.41, H90.42, H90.6, H90.71, H90.72, H90.8, H91.3, H91.01, H91.0, H91.03, H91.09, H91.8X1, H91.8X2, H91.8X3, H91.8X9, H91.90, H91.91, H91.92, H91.93

Abbreviations: CAD, coronary artery disease; CKD, chronic kidney disease; CVA, cerebrovascular accident; HF, heart failure; ICD-9-CM, International Classification of Diseases, 9th Revision, Clinical Modification; ICD-10-CM, International Classification of Diseases, 10th Revision, Clinical Modification

© 2024 Pan HC et al. *JAMA Network Open.*

eTable 2. Medication codes used in this study

Medicine		ATC codes
Hypertension	ACEi	C09A, C09B
	ARB	C09C, C09D
	Alpha-adrenergic blocker	C02CA
	Beta blocking agents	C07
	CCB	C08
	Diuretics	C03
	Others	C02, C04
Anti-uric acid	Probenecid	M04AB01
	Benzbromarone	M04AB03
	Sulfinpyrazone	M04AB02
	Allopurinol	M04AA01
	Febuxostat	M04AA03
	Colchicine	M04AC01
Antiplatelet	Warfarin	B01AA03
	Heparin	B01AB01
	Dalteparin	B01AB04
	Albumin	B05AA01
	Plavix(Clopidogrel)	B01AC04
	Ticlopidine	B01AC05
Statin		C10AA

Abbreviations: ACEi, angiotensin converting enzyme inhibitor; ARB, angiotensin II receptor blocker; CCB, calcium channel blocker;

© 2024 Pan HC et al. *JAMA Network Open.*

eTable 3. Baseline characteristics of enrolled patients and excluded patients

	Total patient (n=22232)	Enrolled patients (n=6703)	Excluded patients (n=15529)	P*
**Demographic factors**				
Age, years	67.5 ± 15.0	68.0 ± 14.7	67.2 ± 14.4	< 0.001
Gender (male), n (%)	12484 (56.2%)	3846 (57.4%)	8638 (55.6%)	0.016
CCI score	4.6 ± 2.3	4.2 ± 2.5	4.8 ± 2.1	< 0.001
Hypertension, n (%)	16641 (74.9%)	4527 (67.5%)	12114 (78.0%)	< 0.001
Diabetes mellitus, n (%)	9573 (43.1%)	2750 (41.0%)	6823 (43.9%)	< 0.001
Myocardial infarction, n (%)	2058 (9.3%)	684 (10.2%)	1374 (8.9%)	0.001
Congestive heart failure, n (%)	7701 (34.6%)	2042 (30.5%)	5659 (36.4%)	< 0.001
Hyperlipidemia, n (%)	6681 (30.1%)	1945 (29.0%)	4736 (30.5%)	0.027
Hyperuricemia, n (%)	2926 (13.2%)	828 (12.4%)	2098 (13.5%)	0.019
Cerebrovascular disease, n (%)	3696 (16.6%)	1125 (16.8%)	2571 (16.6%)	0.676
Malignancy, n (%)	2872 (12.9%)	1190 (17.8%)	1682 (10.8%)	< 0.001
COPD, n (%)	2761 (12.4%)	961 (14.3%)	1800 (11.6%)	< 0.001
Baseline eGFR	32.1 ± 38.7	52.9 ± 43.8	23.1 ± 32.4	< 0.001
Baseline kidney function				< 0.001
CKD stage 0-2	3705 (16.7%)	2305 (34.4%)	1400 (9.0%)	
CKD stage 3	3442 (15.5%)	1856 (27.7%)	1586 (10.2%)	
CKD stage 4	4695 (21.1%)	1512 (22.6%)	3183 (20.5%)	
CKD stage 5	10390 (46.7%)	1030 (15.4%)	9360 (60.3%)	
**Intervention during index hospitalization**				

© 2024 Pan HC et al. *JAMA Network Open.*

Hospitalization, days	29.2 ± 33.8	28.4 ± 27.7	29.5 ± 36.2	0.025
ICU admission, n (%)	12316 (55.4%)	4875 (72.7%)	7441 (47.9%)	< 0.001
Oxygen therapy, n (%)	19475 (87.6%)	6098 (91.0%)	13377 (86.1%)	< 0.001
MV, n (%)	6376 (28.9%)	2929 (43.7%)	3447 (22.2%)	< 0.001
Prolonged MV > 24 hrs, n (%)	5611 (25.2%)	2749 (41.0%)	2862 (18.4%)	< 0.001
ARDS, n (%)	678 (3.1%)	328 (4.9%)	350 (2.3%)	< 0.001
CABG, n (%)	299 (1.3%)	135 (2.0%)	164 (1.1%)	< 0.001
PTCA, n (%)	1365 (6.1%)	432 (6.4%)	933 (6.0%)	0.213
IABP, n (%)	328 (1.5%)	187 (2.8%)	141 (0.9%)	< 0.001
ECMO, n (%)	235 (1.1%)	158 (2.4%)	77 (0.5%)	< 0.001
Major operation				
Cardiac surgery, n (%)	910 (4.1%)	476 (7.1%)	434 (2.8%)	< 0.001
Thoracic surgery, n (%)	607 (2.7%)	266 (4.0%)	341 (2.2%)	< 0.001
Aorta surgery, n (%)	155 (0.7%)	93 (1.4%)	62 (0.4%)	< 0.001
Esophagus surgery, n (%)	103 (0.5%)	52 (0.8%)	51 (0.3%)	< 0.001
Gastric surgery, n (%)	132 (0.6%)	64 (1.0%)	68 (0.4%)	0.0026
Intestine surgery, n (%)	271 (1.2%)	122 (1.8%)	149 (1.0%)	0.039
Rectum surgery, n (%)	73 (0.3%)	30 (0.5%)	43 (0.3%)	0.022
Liver surgery, n (%)	179 (0.8%)	111 (1.7%)	68 (0.4%)	0.008
Biliary surgery, n (%)	103 (0.5%)	52 (0.8%)	51 (0.3%)	0.0113
Pancreas surgery, n (%)	25 (0.1%)	17 (0.3%)	8 (0.1%)	< 0.001
**AKI contributor**				
Sepsis dominant, n (%)	6252 (28.1%)	2803 (41.8%)	3449 (22.2%)	< 0.001
Hypovolemic shock dominant, n (%)	356 (1.6%)	165 (2.5%)	191 (1.2%)	< 0.001

© 2024 Pan HC et al. *JAMA Network Open.*

Contrast dominant, n (%)	4590 (20.7%)	1663 (24.8%)	2927 (18.9%)	< 0.001
Other or mixed causes*, n (%)	15015 (67.5%)	4114 (61.4%)	10901 (70.2%)	< 0.001
**Medication before index hospitalization**				
Antiplatelet, n (%)	2540 (11.4%)	836 (12.5%)	1704 (11.0%)	0.001
Statin, n (%)	8556 (38.5%)	2448 (36.5%)	6108 (39.3%)	< 0.001
Urate-lowering drug, n (%)	8824 (39.7%)	2237 (33.4%)	6587 (47.4%)	< 0.001
Alpha-blocker, n (%)	568 (2.6%)	107 (1.6%)	461 (3.0%)	< 0.001
Beta-blocker, n (%)	3196 (14.4%)	718 (10.7%)	2478 (16.0%)	< 0.001
ACEI or ARB, n (%)	1571 (7.1%)	449 (6.7%)	1122 (7.2%)	0.160
MRA, n (%)	702 (3.2%)	248 (3.7%)	454 (2.9%)	0.0002
CCB, n (%)	3832 (17.2%)	928 (13.8%)	2904 (18.7%)	< 0.001
Other anti-hypertensives, n (%)	595 (2.7%)	136 (2.0%)	459 (3.0%)	< 0.001
Discharge BUN	38.9 ± 23.5	37.0 ± 25.2	39.6 ± 23.1	< 0.001
Discharge eGFR	35.7 ± 44.2	55.5 ± 61.4	27.2 ± 30.4	< 0.001
**Outcome**, n (%)				< 0.001
All-cause Mortality	5787 (26.0%)	1899 (28.3%)	3888 (25.0%)	< 0.001
MACE	3462 (15.6%)	746 (11.1%)	2513 (16.2%)	< 0.001
ESKD	9430 (42.4%)	1119 (16.7%)	8311 (53.5%)	< 0.001
Re-admission	16861 (75.8%)	4333 (64.6%)	11613 (74.8%)	< 0.001

Abbreviations: ACEI, angiotensin converting enzyme inhibitors**;** AKD, acute kidney disease; AKI, acute kidney injury; ARB, angiotensin receptor blockers; ARDS, acute respiratory distress syndrome**;** CABG, coronary artery bypass graft; CCB, *calcium* channel blocker**;** CCI, Charlson Comorbidity Index; CKD, chronic kidney disease; COPD, chronic obstructive pulmonary disease; CT, computerized tomography; ECMO, extra-corporeal membrane oxygenation***;*** ESKD, end-stage kidney disease; IABP, intra-aortic balloon pump**;** ICU, intensive care unit; MACE, major adverse cardiac event; MRA,

© 2024 Pan HC et al. *JAMA Network Open.*

mineralocorticoid receptor antagonists; MV, mechanical ventilation; PMV, persistent mechanical ventilation; PTCA, percutaneous transluminal coronary angioplasty

* Other or mixed causes: nephrotoxic agent, cardiogenic shock, cardiorenal syndrome, obstructive uropathy, hypertension crisis, postpartum, etc.

* Analysis of Variance, ANOVA

© 2024 Pan HC et al. *JAMA Network Open.*

eTable 4. Cox proportional hazards models for adverse outcomes, fitting models with respect to baseline kidney function, AKD severity, and post-AKD kidney function (separate models for each exposure)

	All-cause mortality		MACE*		ESKD*		Readmission*	
**Predictors**	HR with 95%CI	p-value	sHR with 95%CI	p-value	sHR with 95%CI	p-value	sHR with 95%CI	p-value
**Baseline kidney function[&]**								
**CKD Stage 3**	0.98 (0.87 – 1.11)	0.734	1.56 (1.24 – 1.96)	< 0.001	4.61 (3.11 – 6.86)	< 0.001	0.96 (0.88 – 1.04)	0.334
**CKD stage 4**	0.95 (0.83 – 1.08)	0.419	2.07 (1.64 – 2.62)	< 0.001	18.95 (13.02 – 27.58)	< 0.001	1.12 (1.02 – 1.23)	0.013
**CKD stage 5**	0.76 (0.64 - 0.89)	0.001	1.95 (1.50 – 2.53)	< 0.001	45.05 (30.97 – 65.54)	< 0.001	1.19 (1.07 – 1.31)	0.001
**AKD severity[#]**								
**AKD stage 1**	1.03 (0.89 – 1.19)	0.689	0.99 (0.79 – 1.24)	0.945	1.68 (1.44 – 1.97)	<0.001	1.07 (0.98 – 1.18)	0.144
**AKD stage 2**	1.20 (0.96 – 1.49)	0.103	1.09 (0.76 - 1.58)	0.629	1.87 (1.46 – 2.39)	<0.001	1.10 (0.95 - 1.27)	0.202
**AKD stage 3**	0.98 (0.68 – 1.43)	0.931	0.58 (0.25 – 1.29)	0.181	1.47 (0.93 – 2.33)	0.097	1.17 (0.93- 1.48)	0.181
**Post-AKD kidney function[¶]**								
**CKD stage 3**	1.10 (0.96 - 1.27)	0.182	1.69 (1.29 - 2.21)	< 0.001	26.60 (3.63 – 194.91)	0.001	1.03 (0.94 - 1.13)	0.511
**CKD stage 4**	1.31 (1.13 - 1.52)	< 0.001	1.98 (1.49 - 2.62)	< 0.001	192.55 (26.90 – 1378.1)	< 0.001	1.19 (1.08 - 1.32)	0.001
**CKD stage 5**	1.10 (0.94 - 1.28)	0.245	2.52 (1.91 – 3.33)	< 0.001	1013.1 (142.06 –7224.7)	< 0.001	1.53 (1.39 – 1.69)	< 0.001

Abbreviations: AKD, acute kidney disease; AKI, acute kidney injury; CKD, chronic kidney disease; ESKD, end-stage kidney disease; sHR, sub-distribution hazard ratio, HR, hazard ratio

[&] relative to the risk of eGFR > 60 ml/min/1.73m^2

[#] relative to the risk of non-AKD

[¶] relative to the risk of eGFR> 60 ml/min/1.73m^2

*taking mortality as a competing risk.

© 2024 Pan HC et al. *JAMA Network Open.*

eTable 5. Correlation between baseline kidney function, AKD severity, and post-AKD kidney function (Spearman correlation coefficients: r)

	Baseline CKD stage	AKD ratio	Post-AKD CKD stage
Baseline CKD stage	-	0.087**	0.724**
AKD severity	0.087**	-	-0.291**
Post-AKD CKD stage	0.724**	-0.291**	-

Abbreviations: AKD, acute kidney disease; CKD, chronic kidney disease.

* P < 0.05

** P < 0.01

** P < 0.001

© 2024 Pan HC et al. *JAMA Network Open.*

eTable 6. Patient distribution according to the combination of baseline kidney function and post-AKD kidney function

Total number (%)	Post-AKD CKD stage 0-2 (n=1572)	Post-AKD CKD stage 3 (n=1818)	Post-AKD CKD stage 4 (n=1483)	Post-AKD CKD stage 5 (n=1830)
**Baseline CKD stage 0-2**	1266 (54.9%)	784 (34.0%)	175 (7.6%)	80 (3.5%)
**Baseline CKD stage 3**	42 (2.8%)	147 (9.7%)	628 (41.5%)	695 (46.0%)
**Baseline CKD stage 4**	34 (3.3%)	43 (4.2%)	77 (7.5%)	876 (85.0%)
**Baseline CKD stage 5**	42 (2.8%)	147 (9.7%)	628 (41.5%)	695 (46.0%)

Abbreviations: AKD, acute kidney disease; CKD chronic kidney disease

© 2024 Pan HC et al. *JAMA Network Open.*

eTable 7. **Sensitivity analysis for risk of mortality among different post-AKI-CKD stages**

	Post-AKD kidney function					
	CKD stage 3*		CKD stage 4*		CKD stage 5*	
	HR (95% CI)	P	HR (95% CI)	P	HR (95% CI)	P
Original model (n=6703)	1.19 (1.02-1.38)	0.029	1.58 (1.32-1.89)	< 0.001	1.56 (1.26 -1.93)	< 0.001
Eligible subjects with PS for multiple treatments (n=6703)	1.16(0.95-1.42)	0.154	1.62(1.28-2.06)	< 0.001	1.93(1.47-2.53)	< 0.001
Cox regression models with different covariates (n=6703)						
Model 1 (age and gender)	1.10(0.96-1.26)	0.187	1.34(1.16-1.54)	< 0.001	1.05(0.91-1.21)	0.475
Model 2 (age, gender, baseline kidney function and AKD severity)	1.21(1.04-1.41)	0.014	1.68(1.40-2.00)	< 0.001	1.62(1.31-2.00)	< 0.001
Model 3 (age, gender, co-morbidities, baseline kidney function and AKD severity)	1.16(1.00-1.35)	0.055	1.54(1.28-1.84)	< 0.001	1.51(1.22-1.86)	< 0.001
Overlap weighting with different populations (n=5602)						
Model with 180-day landmark analysis[#]	1.24(1.00-1.55)	0.055	1.66(1.27-2.18)	<0.001	1.55(1.13-2.14)	0.007

* Reference: CKD stage 0-2

[#] followed the remaining patients for an additional 180 days from the last day of AKD diagnosis.

Abbreviations: AKD, acute kidney disease; AKI, acute kidney injury; CI, confidence interval; CKD, chronic kidney disease; HR, hazard ratio; PS, propensity score

© 2024 Pan HC et al. *JAMA Network Open.*

eTable 8. **Sensitivity analysis for risk of incident MACE or mortality among different post-AKI-CKD stages**

	Post-AKD kidney function					
	CKD stage 3*		CKD stage 4*		CKD stage 5*	
	HR (95% CI)	P	HR (95% CI)	P	HR (95% CI)	P
Original model (n=6703)	1.24 (1.08 -1.42)	0.003	1.52 (1.29- 1.79)	< 0.001	1.68 (1.40 -2.02)	< 0.001
Eligible subjects with PS for multiple treatments (n=6703)	1.10(0.89-1.35)	0.387	1.34 (1.06-1.71)	0.016	1.72(1.33-2.24)	<0.001
Cox regression models with different covariates (n=6703)						
Model 1 (age and gender)	1.22(1.08-1.40)	0.002	1.51 (1.33-1.72)	< 0.001	1.42(1.25-1.62)	< 0.001
Model 2 (age, gender, baseline kidney function and AKD severity)	1.28(1.11-1.47)	0.001	1.69 (1.44-2.00)	< 0.001	1.88(1.56-2.27)	< 0.001
Model 3 (age, gender, co-morbidities, baseline kidney function and AKD severity)	1.23(1.06-1.41)	0.005	1.52 (1.29-1.80)	< 0.001	1.69(1.39-2.05)	< 0.001
Overlap weighting with different populations (n=5602)						
Model with 180-day landmark analysis[#]	1.22(1.00-1.49)	0.048	1.58(1.25-2.01)	< 0.001	1.77(1.34-2.32)	< 0.001

* Reference: CKD stage 0-2

[#] followed the remaining patients for an additional 180 days from the last day of AKD diagnosis.

Abbreviations: AKD, acute kidney disease; AKI, acute kidney injury; CI, confidence interval; CKD, chronic kidney disease; HR, hazard ratio; MACE, major adverse cardiovascular events; PS, propensity score

© 2024 Pan HC et al. *JAMA Network Open.*

eTable 9. **Cox proportional hazards models depicting the possibility of all-cause mortality, MACE*, ESKD*, and readmission with discharge date as the time zero point.**

	All-cause mortality		MACE		ESKD		Readmission	
**Predictors**	HR with 95%CI	p-value	SHR with 95%CI	p-value	SHR with 95%CI	p-value	SHR with 95%CI	p-value
**Baseline kidney function[&]**								
**CKD Stage 3**	0.81 (0.75 – 0.86)	< 0.001	1.43 (1.24 – 1.66)	< 0.001	2.02 (1.72 – 2.37)	< 0.001	1.00 (0.94 – 1.06)	0.974
**CKD stage 4**	0.72 (0.67 - 0.78)	< 0.001	1.60 (1.38 – 1.86)	< 0.001	3.90 (3.33 – 4.57)	< 0.001	1.07 (1.01 – 1.14)	0.035
**CKD stage 5**	0.49 (0.46 - 0.54)	< 0.001	1.66 (1.43 - 1.93)	< 0.001	5.10 (4.36 – 5.98)	< 0.001	0.92 (0.87 - 0.98)	0.011
**Discharge kidney function[¶]**								
**CKD stage 3**	1.15 (1.04 - 1.26)	0.007	1.49 (1.22 – 1.83)	0.001	2.39 (1.79 – 3.20)	< 0.001	1.00 (0.93 - 1.08)	0.931
**CKD stage 4**	1.52 (1.38 - 1.68)	< 0.001	1.63 (1.33 – 1.99)	< 0.001	4.70 (3.54 – 6.24)	< 0.001	1.14 (1.06 - 1.22)	< 0.001
**CKD stage 5**	1.51 (1.36 - 1.66)	< 0.001	1.47 (1.20 – 1.79)	0.001	6.93 (5.22 –9.20)	< 0.001	1.20 (1.12 – 1.29)	< 0.001

Abbreviations: AKD, acute kidney disease; AKI, acute kidney injury; CKD, chronic kidney disease; ESKD, end-stage kidney disease; HR, hazard ratio

[&] relative to the risk of eGFR > 60 ml/min/1.73m^2

[¶] relative to the risk of eGFR> 60 ml/min/1.73m^2

*taking mortality as a competing risk.

© 2024 Pan HC et al. *JAMA Network Open.*

eTable 10. Cause-specific Cox models depicting the possibility of all-cause mortality, MACE, ESKD, and readmission

	All-cause mortality		MACE		ESKD		Readmission	
**Predictors**	HR with 95%CI	p-value	HR with 95%CI	p-value	HR with 95%CI	p-value	HR with 95%CI	p-value
**Baseline kidney function**[&]								
**CKD Stage 3**	0.84 (0.73 – 0.97)	0.016	1.31 (1.02 – 1.68)	0.035	2.68 (1.77 – 4.04)	< 0.001	0.88 (0.80 – 0.96)	0.006
**CKD stage 4**	0.72 (0.60 - 0.86)	< 0.001	1.49 (1.10 – 2.00)	0.009	5.08 (3.35 – 7.71)	< 0.001	0.84 (0.75 – 0.95)	0.004
**CKD stage 5**	0.57 (0.45 - 0.71)	< 0.001	1.24 (0.87 - 1.77)	0.225	8.80 (5.71 – 13.56)	< 0.001	0.78 (0.68 - 0.90)	0.001
**AKD severity**[#]								
**AKD stage 1**	0.92 (0.80 – 1.07)	0.301	0.93 (0.74 – 1.18)	0.560	1.75 (1.48 – 2.07)	<0.001	0.98 (0.89 – 1.08)	0.670
**AKD stage 2**	1.01 (0.81 – 1.27)	0.931	1.02 (0.70 - 1.49)	0.914	2.25 (1.72 – 2.93)	<0.001	0.96 (0.82 - 1.12)	0.577
**AKD stage 3**	0.73 (0.49 – 1.09)	0.122	0.52 (0.23 – 1.20)	0.124	2.19 (1.32 – 3.62)	0.002	0.91 (0.72- 1.17)	0.469
**Post-AKD kidney function**[¶]								
**CKD stage 3**	1.19 (1.02 - 1.38)	0.029	1.54 (1.15 - 2.06)	0.003	17.95 (2.44 – 132.10)	0.005	1.08 (0.98 - 1.20)	0.115
**CKD stage 4**	1.58 (1.32 - 1.89)	< 0.001	1.63 (1.17 - 2.28)	0.004	82.36 (11.40 – 565.15)	< 0.001	1.33 (1.18 - 1.50)	< 0.001
**CKD stage 5**	1.56 (1.26 - 1.93)	< 0.001	2.14 (1.48 – 3.09)	< 0.001	272.25 (37.65 –1968.76)	< 0.001	1.80 (1.56 – 2.07)	< 0.001

Abbreviations: AKD, acute kidney disease; AKI, acute kidney injury; CKD, chronic kidney disease; ESKD, end-stage kidney disease; sHR, sub-distribution hazard ratio, HR, hazard ratio

[&] relative to the risk of eGFR > 60 ml/min/1.73m^2

[#] relative to the risk of non-AKD

[¶] relative to the risk of eGFR> 60 ml/min/1.73m^2

© 2024 Pan HC et al. *JAMA Network Open.*

eTable 11. Cause-specific Cox models depicting the possibility of all-cause mortality, MACE, ESKD, and readmission with discharge date as the time zero point.

	All-cause mortality		MACE		ESKD		Readmission	
**Predictors**	HR with 95%CI	p-value	HR with 95%CI	p-value	HR with 95%CI	p-value	HR with 95%CI	p-value
**Baseline kidney function**[&]								
**CKD Stage 3**	0.81 (0.75 – 0.86)	< 0.001	1.33 (1.14 – 1.54)	0.002	1.85 (1.57 – 2.16)	< 0.001	0.93 (0.88 – 0.99)	0.014
**CKD stage 4**	0.72 (0.67 - 0.78)	< 0.001	1.41 (1.21 – 1.64)	< 0.001	3.33 (2.86 – 3.88)	< 0.001	0.93 (0.87 – 0.98)	0.011
**CKD stage 5**	0.49 (0.46 - 0.54)	< 0.001	1.35 (1.16 - 1.57)	0.001	4.03 (3.46 – 4.68)	< 0.001	0.76 (0.72 - 0.81)	< 0.001
**Discharge kidney function**[¶]								
**CKD stage 3**	1.15 (1.04 - 1.26)	0.007	1.55 (1.26 – 1.90)	< 0.001	2.45 (1.84 – 3.27)	< 0.001	1.06 (0.99 - 1.14)	0.125
**CKD stage 4**	1.52 (1.38 - 1.68)	< 0.001	1.84 (1.51 – 2.25)	< 0.001	5.37 (4.08 – 7.06)	< 0.001	1.30 (1.21 - 1.39)	< 0.001
**CKD stage 5**	1.51 (1.36 - 1.66)	< 0.001	1.65 (1.35 – 2.02)	< 0.001	8.17 (6.22 –10.73)	< 0.001	1.42 (1.32 – 1.53)	< 0.001

Abbreviations: AKD, acute kidney disease; AKI, acute kidney injury; CKD, chronic kidney disease; ESKD, end-stage kidney disease; HR, hazard ratio

[&] relative to the risk of eGFR > 60 ml/min/1.73m^2

[¶] relative to the risk of eGFR> 60 ml/min/1.73m^2

*taking mortality as a competing risk.

© 2024 Pan HC et al. *JAMA Network Open.*

eTable 12. **Specificity analysis for risk of 3 independent events, appendicitis, traffic accident, and deafness among different baseline CKD stages**

	Baseline kidney function					
	CKD stage 3*		CKD stage 4*		CKD stage 5*	
Incident events	HR (95% CI)	P	HR (95% CI)	P	HR (95% CI)	P
Appendicitis (n=6703)	0.62 (0.14- 2.80)	0.536	2.61 (0.58 - 11.63)	0.210	3.06 (0.50-18.70)	0.226
Traffic accident (n=6703)	0.71 (0.16-3.17)	0.650	0.42 (0.05-3.73)	0.436	0.41 (0.03-6.66)	0.530
Deafness (n=6703)	0.95 (0.49-1.85)	0.882	1.25 (0.56-2.83)	0.585	1.27 (0.48-3.36)	0.636

* Reference: CKD stage 0-2

Abbreviations: AKD, acute kidney disease; AKI, acute kidney injury; CI, confidence interval; CKD, chronic kidney disease; HR, hazard ratio

© 2024 Pan HC et al. *JAMA Network Open.*

eTable 13. **Specificity analysis for risk of 3 independent events, appendicitis, traffic accident, and deafness among different AKD stages**

	AKD stages					
	AKD stage 1*		AKD stage 2*		AKD stage 3*	
Incident events	HR (95% CI)	P	HR (95% CI)	P	HR (95% CI)	P
Appendicitis (n=6703)	0.32 (0.03 - 2.96)	0.315	1.21 (0.13 - 10.95)	0.866	4.72 (0.47-47.12)	0.186
Traffic accident (n=6703)	0.41 (0.05-3.69)	0.425	0.00 (0.00-0.00)	1.000	3.12 (0.26-37.32)	0.368
Deafness (n=6703)	1.52 (0.83-2.78)	0.176	1.06 (0.31-3.53)	0.929	2.61 (0.73- 9.32)	0.138

* Reference: No AKD

Abbreviations: AKD, acute kidney disease; AKI, acute kidney injury; CI, confidence interval; CKD, chronic kidney disease; HR, hazard ratio

© 2024 Pan HC et al. *JAMA Network Open.*

eTable 14. **Specificity analysis for risk of 3 independent events, appendicitis, traffic accident, and deafness among different post-AKI CKD stages**

	Post-AKD kidney function					
	CKD stage 3*		CKD stage 4*		CKD stage 5*	
Incident events	HR (95% CI)	P	HR (95% CI)	P	HR (95% CI)	P
Appendicitis (n=6703)	1.06 (0.28 – 4.03)	0.935	0.38 (0.06-2.33)	0.298	0.40 (0.06-2.72)	0.350
Traffic accident (n=6703)	1.60 (0.33-7.69)	0.560	1.60 (0.19-13.12)	0.663	1.05 (0.09-12.62)	0.970
Deafness (n=6703)	0.85 (0.43-1.70)	0.653	0.59 (0.25-1.41)	0.239	0.70 (0.27-1.83)	0.468

* Reference: CKD stage 0-2

Abbreviations: AKD, acute kidney disease; AKI, acute kidney injury; CI, confidence interval; CKD, chronic kidney disease; HR, hazard ratio

© 2024 Pan HC et al. *JAMA Network Open.*
