# Supplementary material for: Recovery Dynamics and Prognosis After Dialysis for Acute Kidney Injury
Source: JAMA Netw Open. 2024 Mar 8;7(3):e240351. doi: 10.1001/jamanetworkopen.2024.0351 (PMC10924241; doi:10.1001/jamanetworkopen.2024.0351)
Supplement: Supplement 2. — Data Sharing Statement [file jamanetwopen-e240351-s002.pdf]

Data Sharing Statement

Pan. Baseline Kidney Function and Recovery Dynamics After Acute Kidney Injury Requiring Dialysis. *JAMA Netw Open*. Published March 08, 2024.
doi:10.1001/jamanetworkopen.2024.0351

Data
Data available: No

Additional Information
Explanation for why data not available: The datasets used and/or analyzed during the current study are available from the corresponding author on reasonable request. However, we do not have individual patient data.
